# Probing Solvent Dynamics at the Single-Molecule Limit

**DOI:** 10.1021/acs.nanolett.6c03272

**Published:** 2026-09-22

**Authors:** Roberto Listo, James M. F. Morris, Amit Sil, Xiaohang Qiao, R. Tom Abram, Chanyuan Huo, Jonathan S. Ward, Walther Schwarzacher, Piero Malcovati, Marco Vacca, Mariagrazia Graziano, Giovanna Turvani, Richard J. Nichols, Andrea Vezzoli

**Affiliations:** † Donnan Laboratories, Department of Chemistry, 4591 University of Liverpool, Crown Street, Liverpool L69 7ZD, United Kingdom; ‡ Department of Electrical, Computer and Biomedical Engineering, University of Pavia, Pavia 27100, Italy; § Department of Electronics and Telecommunications, 19032 Politecnico di Torino, Torino 10129, Italy; ∥ H.H. Wills Physics Laboratory, University of Bristol, Tyndall Avenue, Bristol BS8 1TL, United Kingdom; ⊥ Department of Chemistry, 4396 Lancaster University, Lancaster LA1 4YB, United Kingdom; # Syed Babar Ali School of Science and Engineering, 66687 Lahore University of Management Sciences, 54792 Lahore, Pakistan; ∇ Department of Applied Science and Technology, Politecnico di Torino, Torino 10129, Italy

**Keywords:** Single-Molecule Junctions, Solvent Effects, Environmental Effects, Single-Molecule Conductance, Alternate-Current Electrochemical Dynamics

## Abstract

The Electric Double Layer (EDL) that self-organizes at electrode–electrolyte interfaces plays a fundamental role in electrochemistry. While its structure has been extensively studied over the last 150 years, its *dynamic* behavior is still not completely understood. Here, we present a novel experimental approach to probe the EDL using single-molecule junctions as ultrasensitive probes, validated by a preliminary study of the Inner Helmholtz Plane (IHP). We measured the current response of single-molecule devices under square-wave bias in various solvent environments, and we found remarkable differences falling into three categories: large nonlinear transients in highly polar solvents, capacitor-like responses in solvents of intermediate polarity, and no significant transients (within our frequency limits) in nonpolar solvents. By fitting the solvent behavior to a molecular-level equivalent circuit, our data reveal trends in how the nature of the solvent influences IHP dynamics and provide insights into the origin of nonlinearity.

## Graphical abstract

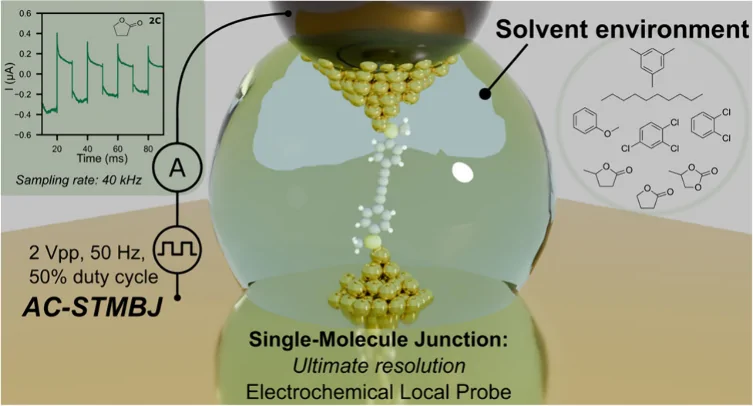

The electric double layer (EDL) at electrified interfaces governs charge screening and ionic organization under applied electric fields, and it plays a central role in processes ranging from energy storage and conversion to nanoscale charge transport.^1,2^ Understanding the structure and dynamic behavior of the EDL is increasingly crucial in the context of single-molecule junctions (SMJs), where evidence indicates that solvents, electrolytic environments and ion dynamics can strongly influence charge transport. The environment of the junction has been specifically exploited to impart functionality, ranging from rectification^3,4^ to their use as quantum probes^5,6^ and sensors,^7^ going beyond their enabling role in more traditional electrochemical gating of molecular transport.^8–10^ Furthermore, environmental effects underpin key mechanisms of emergent neuromorphic behaviors in molecular devices, where ionic motion and EDL restructuring contribute to memory, hysteresis, and synaptic-like responses.^2,11^ From an electrochemical perspective, the dynamic response of the EDL to time-dependent electric fields remains far less understood, especially at the nanometre and subnanometre length scales relevant to molecular and atomic-scale interfaces.^12^ Most experimental approaches to EDL dynamics rely on bulk spatial ensemble-averaged measurements, which provide valuable information on average charging kinetics but obscure local heterogeneity and interfacial fluctuations.^13,14^ Recent nanoscale and time-resolved studies have sought to overcome these limitations by probing EDL dynamics with electrochemical impedance spectroscopy implemented within a scanning tunnelling microscope platform, which has been used to quantify the capacitance of the dipole layer formed at the metal–solvent interface.^15^ However, the influence of solvent *dynamics* on a single conductance channel has remained largely unexplored.

Probing EDL dynamics at the molecular scale requires experimental platforms that combine alternating electric-field excitation, nanometre spatial confinement, and frequency or time-resolved electrical readout. SMJs naturally meet these requirements, as the applied bias is confined to subnanometre distances, generating local electric fields of up to several volts per nanometre while providing direct access to charge transport through an individual molecular channel.^16,17^ In this framework, the scanning tunnelling microscope break-junction (STMBJ) technique^18^ offers a statistically robust methodology to form metal–molecule– metal junctions in liquid environments while probing their electrical response under controlled AC bias conditions (AC-STMBJ).^19,20^ Although SMJs have been extensively employed to study molecular charge transport, rectification, and gating phenomena, their use as local probes of interfacial electrochemical dynamics remains largely unexplored.^21,22^ Nevertheless, the solvent environment alone has been shown to affect charge transport in single-molecule devices, demonstrating their unique sensitivity to the local electrostatics.^15,23,24^ The mechanisms involved are chemically specific: solvents adsorb on the electrodes and shift the alignment between the dominant transport orbital and the metal Fermi level,^25^ protic solvents hydrogen-bond to the molecular backbone,^26^ and strongly structured media such as deep eutectic solvents modify the interfacial potential distribution.^27^ Here, we employ the AC-STMBJ technique to directly probe solvent dynamics, providing experimental access to the dynamics of dipole reorganization processes at the solvent-populated Inner Helmholtz Plane (IHP) and to establish a “solvent baseline” for further experiments in electrolytic environments.

Figure 1a shows the SMJ probe employed in this work: a single molecule trapped between two gold electrodes exposed to a solvent environment. We used a bis­(thioanisolyl)­butadiynyl molecular wire (see Figure 1b) as a trade-off between stability, conductance, and ease of synthesis. The simple, rigid structure of the molecular wire and the thioanisolyl termini further ensure the absence of electric-field driven reactivity^17,28,29^ or conformational changes^30–32^ that have been shown to occur in other materials. Junctions were fabricated and characterized using a modified AC-STMBJ technique. In our implementation, the STM Au tip is driven into the Au substrate to form a metallic microcontact and then withdrawn at constant speed (10 *nm s* ^–1^ in this study). As the tip is witdrawn, the microcontact is reduced to a point contact that eventually breaks to deliver a nanogap separated by two atomically sharp electrodes. The experiment is performed in a 100 μ*M* solution of the target molecular wire, so that the molecule can self-assemble in the freshly formed nanogap to yield the molecular junction. Once the junction is in place, we stopped the tip withdrawal process for 100 *ms*. A ± 1 *V* square-wave bias excitation is then applied (i.e., 2 *V*_*pp*_, 50 *Hz*, 50% duty cycle), and the resulting time-dependent current response is acquired at a 40 *kHz* sampling rate. After this process, we further withdraw the tip to rupture the junction, and the process is repeated thousands of times to deliver sufficient statistical robustness. Details about the instrumentation used in this work can be found in our recent publications about the subject,^33,34^ and further details about the specific modifications to our apparatus that enabled the experiments described here are provided in the SI.

**Figure 1:**
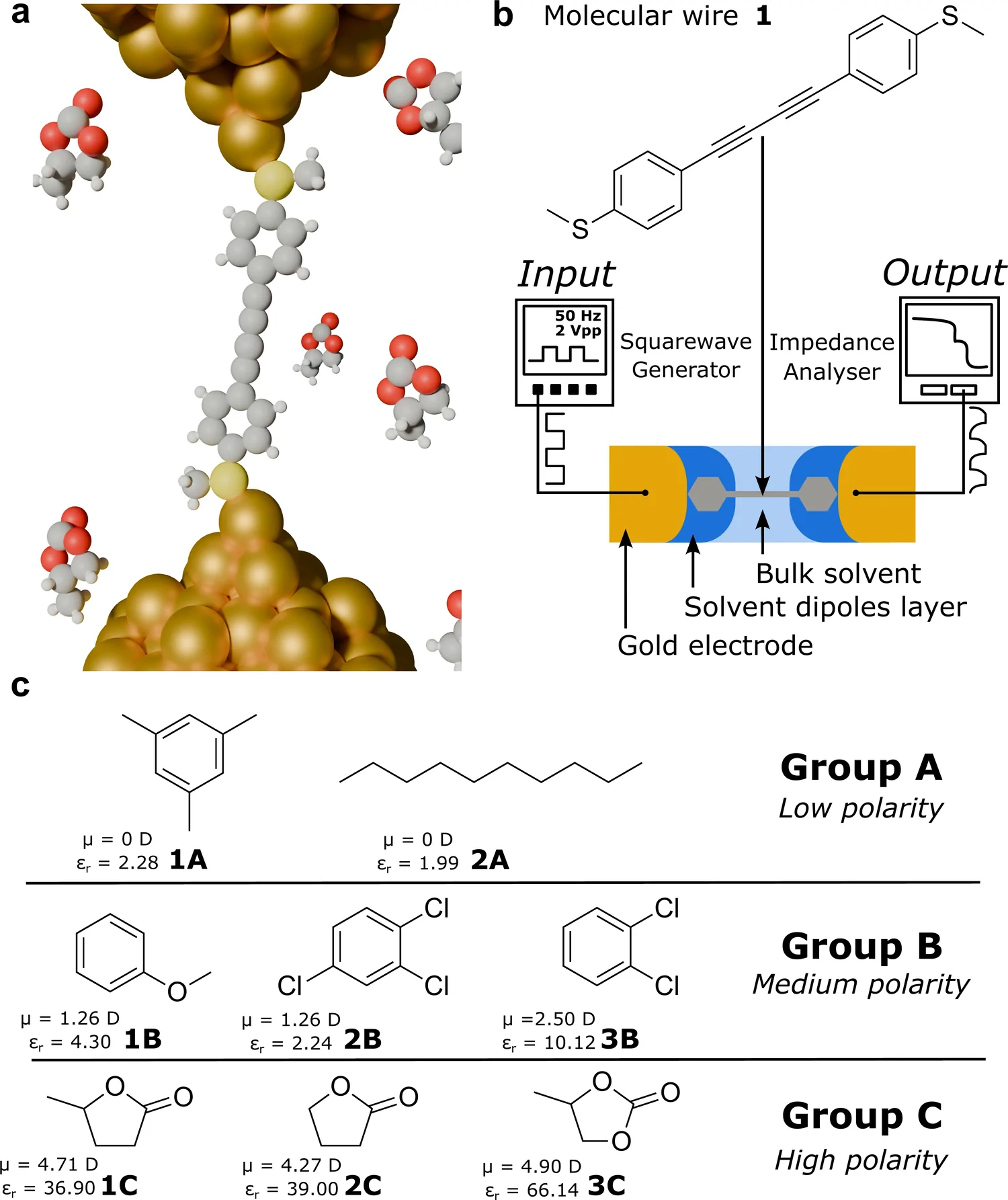
(a) Rendered representation of a representative device configuration, showing a bis­(thioanisolyl)­butadiynyl molecular wire bridging two Au electrodes via coordinative S–Au bonds. A limited number of solvent molecules is included to qualitatively illustrate the local environment. (b) Schematic of the experimental setup employed in this work. A square-wave voltage (2 *V*_*pp*_ i.e. ± 1 *V*, 50 *Hz*) is applied to the single-molecule junction (SMJ), while the output signal reflects time-dependent impedance (current) variations. The SMJ conductance is continuously monitored under different solvent environments. (c) Set of solvents investigated in this study, selected based on polarity in order to span a broad range of physicochemical properties, with the molecular dipole moment μ and the static relative permittivity *ε*_*r*_ reported beneath each structure: **1A** mesitylene, **2A** decane, **1B** anisole, **2B** 1,2,4-trichlorobenzene, **3B** 1,2-dichlorobenzene, **1C** γ-valerolactone, **2C** γ-butyrolactone, and **3C** propylene carbonate. The three panels correspond to **Group A** (low polarity, μ *<* 0.5 *D*, *ε*_*r*_ < 2.5), **Group B** (medium polarity, μ = 1.2 – 2.5 *D*, *ε*_*r*_ = 2.2 – 10.1) and **Group C** (high polarity, μ > 4 *D*, *ε*_*r*_ > 35). Numerical values for all eight solvents are collected in Table S1.

This setup records the transient response following each bias inversion with high temporal resolution. The molecular junction acts as a *nanoscale transducer*, converting local charge redistributions into a measurable electrical signal. After screening 21 solvents, we identified eight that concurrently offer (i) solubilizing power for the chosen molecular wire (ii) high boiling point and low volatility to ensure stable concentration over the data acquisition time frame, (iii) wide enough spread of fundamental physical properties (e.g., dielectric constant, dipole moment, viscosity, etc.), and (iv) compatibility with STMBJ experiments (i.e., not impairing the formation of single-molecule junctions). Figure 1c presents the solvents used in this work, grouped quantitatively by their molecular dipole moment: **Group A** (low polarity, μ < 0.5 D), including mesitylene (**1A**) and decane (**2A**), **Group B** (medium polarity, μ = 1.2–2.5 D), including anisole (**1B**), 1,2,4-trichlorobenzene (**2B**), and 1,2-dichlorobenzene (**3B**), and **Group C** (high polarity, μ > 4 D), including γ-valerolactone (**1C**), γ-butyrolactone (**2C**), and propylene carbonate (**3C**). Corresponding permittivity and viscosity are collated in Table S1. The complete screening campaign, covering twenty-one solvents, is documented in Table S2. For each solvent environment, we performed AC-STMBJ measurements and select the traces corresponding to successful formation of SMJs stable for the entire 100 ms of AC modulation (see SI for data acquisition/processing procedure). The median trace of each data set was evaluated and linearly detrended to minimize the effect of piezoelectric actuator drift during the measurement, enabling accurate fitting to an equivalent circuit model. Figure 2a summarizes the data-processing workflow.^35^

**Figure 2:**
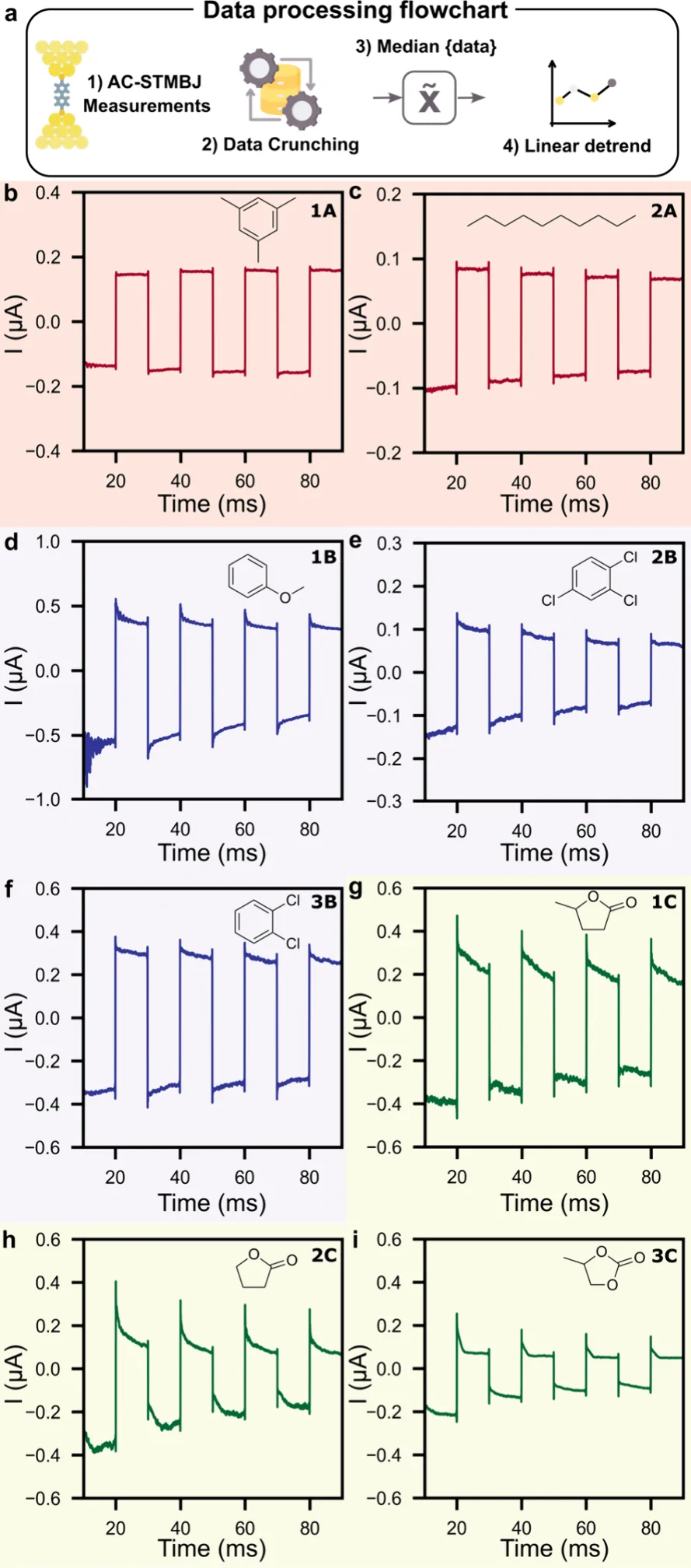
(a) Flowchart of the AC-STMBJ data processing pipeline: acquisition of the raw traces, data crunching, trace-by-trace median extraction, and linear detrending. (b–i) Representative single current traces measured in the investigated solvents: mesitylene, decane, anisole, 1,2,4-trichlorobenzene, 1,2dichlorobenzene, γ-valerolactone, γ-butyrolactone, and propylene carbonate.

Figure 2(b–i) shows the results for each solvent after the selection and processing procedure. Three distinct regimes are identified in the transient response. For **Group A** (nonpolar) solvents, the current traces are insensitive to the applied square-wave excitation, and no transient features are resolved within the experimental bandwidth. On a 25*μs* time scale, no dynamic behavior is experienced: the environment around the molecular junction remains essentially unaltered by the AC bias. In contrast, **Group B** solvents exhibit a clear, reproducible, and symmetric transient, decaying within 10*ms* of the pulse edge and consistent with capacitive charging/discharging associated with solvent dipole reorientation. The temporal evolution of the conductance follows a linear response to the applied field, indicating that interfacial charge rearrangements occur on time scales comparable to the experimental time resolution. A different behavior is observed for the highly polar solvents in **Group C**, which display pronounced nonlinear and asymmetric transients upon positive and negative bias steps. These features point to a strong field-dependent reorganization of the interfacial charge configuration, leading to distinct charging and discharging pathways.

The response observed in **Group A** and **Group B** is rationalized within a molecular-scale, linear time-invariant equivalent circuit. The correspondence between circuit elements and the physical system is illustrated in Figure 3a, and the resulting equivalent circuit is shown in Figure 3b. The molecular junction is described by the resistance *R*_*m*_, the solvent conductivity by the resistance *R*_*s*_, and the interfacial dipolar layers at the metal–solution interfaces by capacitances *C*_1_ and *C*_2_, combined into an effective capacitance *C*_*eq*_. We fit the experimental data to the model parameters to extract *R*_*m*_, *R*_*s*_, and *C*_*eq*_ values for every solvent belonging to **Group A** and **Group B** (see SI for parameter fitting details). Before adopting this circuit, we evaluated three alternative topologies (Section S4.2 of the SI): a Randles circuit including charge-transfer resistances at each interface, an explicit two-capacitor model retaining *C*_1_ and *C*_2_ as independent parameters, and a constant-phase (Cole–Cole) element in place of the ideal interfacial capacitance. The first two are structurally nonidentifiable from a single-pole step response (they add free parameters without adding observables) and formal model selection on the experimental transients favors the three-parameter circuit adopted here over all alternatives.

**Figure 3:**
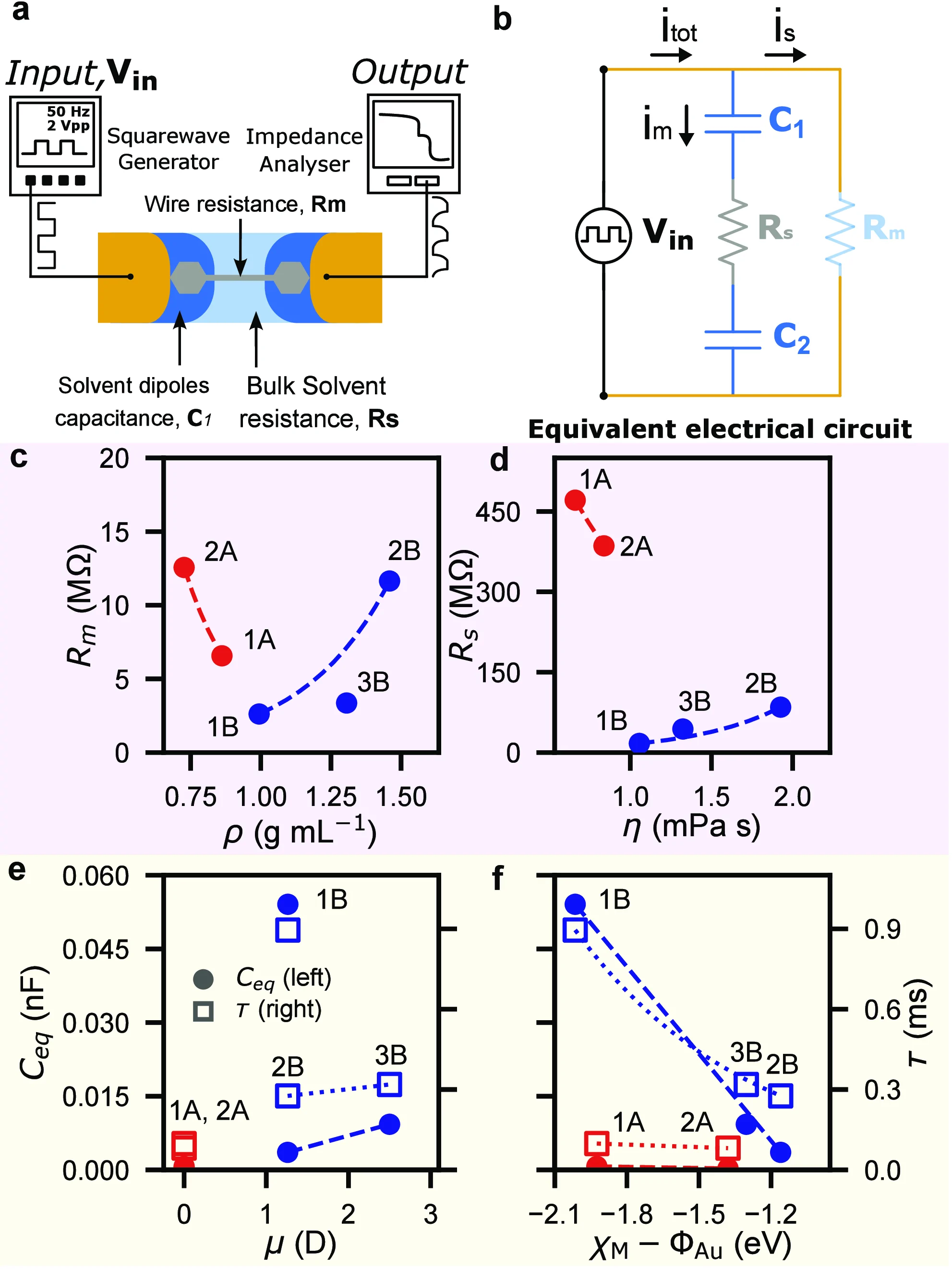
(a) Schematic of the AC-STMBJ experiment, showing the electrical association of each physical element: the molecular wire resistance *R*_*m*_, the interfacial capacitances *C*_1_ and *C*_2_ arising from solvent dipolar layers, and the bulk solvent resistance *R*_*s*_. (b) Equivalent electrical circuit of the SMJ in solution, with the total current dividing between the molecular branch and the interfacial branch. (c) Molecular wire resistance *R*_*m*_ as a function of the solvent density. (d) Solvent resistance *R*_*s*_ (see main text for interpretation of this value) as a function of the solvent viscosity. (e) Equivalent capacitance *C*_*eq*_ (filled circles, left axis) and the time constant τ (open squares, right axis) as a function of the molecular dipole moment. (f) The same two quantities as a function of *Δχ* = *χM* – *ΦAu*, the difference between the Mulliken electronegativity of the solvent molecule and the work function of gold. In panels (c)-(f) red denotes Group A and blue Group B, and solvents are identified by the group codes of Figure 1c. Dashed and dotted lines are Theil-Sen guides to the eye, fitted to each group separately. Panels (c,d) and (e,f) are shaded differently only to separate the resistive from the capacitive parameters.

Figure 3c shows that *R*_m_ exhibits distinct dependences. For **Group A** solvents (negligible dipole moment), *R*_m_ decreases with increasing solvent density. This trend suggests that, in the absence of significant dipolar interactions, increased molecular packing of the solvent around the molecular bridge stabilizes the junction geometry. A more stable junction would enhance electronic coupling at the electrode–molecule interfaces, resulting in lower effective molecular resistance *R*_m_. In contrast, for **Group B** solvents possessing a finite dipole moment, *R*_m_ increases with increasing density. This behavior suggests that denser polar environments enhance electrostatic screening and modify the local potential landscape, effectively increasing the tunnelling barrier sensed by the charge carriers. The opposite trends observed across the two groups indicate that the presence or absence of permanent molecular dipoles opposes the role of the solvent density.

The values of solvent resistance *R*_s_ extracted from our model provide unique insights into two phenomena happening at the single-molecule junction. First, their absolute value demonstrates that this is not the resistance of the “bulk solvent”. Given the conditions employed here, the values obtained should be >1*G*Ω, but values in the > *M*Ω range have been extracted. The latter is compatible with the model of molecular solvation shells (or tightly bound solvent molecules) actively participating to charge transport, introduced to rationalize the unusually high value of conductance observed in proteins.^36^ In this case, the phenomenon is relatively unremarkable in magnitude (contributing only up to 3% of the overall charge transport) but robust enough to be observed. Second, there is a viscosity dependence that differs between the two groups (Figure 3d). For **Group A** solvents, *R*_*s*_ decreases with increasing viscosity, consistent with a scenario in which slower molecular motion reduces dynamic fluctuations at the interface and leads to a more stable (i.e., less resistive) environment. In **Group B** solvents, *R*_*s*_ increases with viscosity, from 17 *MΩ* in anisole to 85 *MΩ* in 1,2,4-trichlorobenzene. The two opposing trends suggest that once permanent dipoles are present, the solvation shell that carries this leakage stiffens as the medium becomes more sluggish, so that a “lower limit” of solvation shell resistance is not reached within the range of viscosities sampled here.

The equivalent capacitance *C*_*eq*_ highlights the distinction between the two solvent classes (Figure 3e,f). For **Group A** *C*_*eq*_ is negligible within experimental uncertainty, consistent with the absence of observable charging and discharging features in the transient response. In contrast, for **Group B** solvents, *C*_*eq*_ is finite and spans more than an order of magnitude across the group, from 36 *pF* in 1,2,4-trichlorobenzene to 54 *pF* in anisole. No bulk property can account for such a variation. Across **Group B** the values of permittivity, viscosity, density, and dipole moment do not order the group at all (see Figure 3e for an example based on μ). Adsorption of solvents on gold is known to reshape the potential distribution across the inner Helmholtz plane,^25^ and a more strongly adsorbed layer presents a thinner and more polarizable interfacial region, and hence a larger capacitance. We attribute the observed phenomena to specific solvent–electrode interactions rather than to bulk polarity. At the solvent-electrode interface, charge flows until the two potentials equalize, driven by the difference between the electronegativity of the solvent molecule and that of the metal. We therefore take *Δχ* = *χM* – Φ*Au* as the abscissa of Figure 3f, where *χM* = (*IP* + *EA*)/2 is the Mulliken (absolute) electronegativity of the solvent molecule and Φ*Au* = 5.31 *eV* is the work function of Au(111). Both *IP* and *EA* are computed for the whole molecule. *Δχ* is negative for every solvent studied here, meaning that charge flows from the solvent toward the metal, and its magnitude of charge transfer sets the size of the interface dipole that develops across the inner Helmholtz plane (the interfacial capacitance in the equivalent circuit). Within **Group B** the three solvents span 0.86 *eV* in *Δχ*, and both *C*_*eq*_ and τ fall monotonically along that sequence (Figure 3f). At this stage, this is a qualitative correlation as three solvents cannot establish a functional form, and the computed electron affinities correspond to anionic resonances rather than true bound states. Within **Group B** *Δχ* ranks the solvents in the same order as the viscosity, so we do not attempt to separate the two contributions. Finally, the relaxation time τ tracks *C*_*eq*_ (Figure 3e,f). For **Group A** solvents, τ is negligible, while for **Group B** it follows the same ordering as the capacitance, falling as *Δχ* approaches zero. This behavior is consistent with the relation τ ∼ *R*_*s*_ *C*_*eq*_ and suggests that the transient response is governed primarily by interfacial charging dynamics. Overall, the data indicate that solvent viscosity and specific the solvent–electrode chemistry define the electrostatic landscape experienced by the SMJ, even in the absence of added ionic species.

Solvents belonging to **Group C** exhibit nonlinear and asymmetric transient responses, which cannot be captured by the linear time-invariant framework discussed above. This is not a shortcoming of the circuit adopted here. A linear time-invariant network driven by a half-wave antisymmetric excitation, *v*(t + *T*/2) = -*v*(t), necessarily produces a periodic steady-state response obeying *i*(*t* + *T*/2) = -*i*(*t*). The asymmetry observed in **Group C** excludes *any* linear equivalent circuit, however many elements it contains, and demands a field-dependent interfacial element instead. We verify this explicitly in Section S4.2.5 of the SI, where we also show that introducing a single field-dependent capacitance is sufficient to generate an asymmetric response. We want to stress that the asymmetry of the electrodes alone – arising from using a coated Au tip to suppress faradaic currents – does not seem to be underpinning the observed asymmetry in electrical response: measurements in 1,2-dichlorobenzene (solvent **3B**) were also performed with a coated tip, but the response is perfectly symmetric (see Figure 2f).^3^

To elucidate the physical origin of this regime, we performed a series of control experiments (Figure 4). To exclude the possibility that the dominant nonlinearity originates from geometric or electronic asymmetries of the metallic electrodes, we performed an initial-state dependence test in γ-butyrolactone. The system is first stabilized under a steady-state hold condition at either +0.2 *V* or −0.2 *V*, followed by the application of an AC excitation (Figure 4a). We distinguish between cases where the first excursion of the AC signal corresponds to either a rising or falling edge with respect to the initial bias. Our results (Figure 4b) reveal a pronounced dependence of the current response on both the initial condition (+0.2 *V* or −0.2 *V*) and the amplitude of the first voltage excursion (+1.2 *V*, −1.2 *V*, or (+0.8 *V*). Notably, when the first excursion from the steady state (independently of the initial bias polarity and whether it corresponds to a rising or falling edge) reaches *ΔV* = 1.2 *V*, the AC conductance remains consistently higher throughout the entire hold phase. This behavior suggests that the initial high-field excursion induces a strong dipolar alignment of the solvent molecules at the interface. Once established, this ordered configuration persists over time without undergoing subsequent reorientation, consistent with a field-induced locking mechanism. Such a scenario is in line with the concept of electro-freezing, where cooperative dipole–field interactions lead to long-lived, ordered interfacial structures that suppress further reconfiguration.^37^ Hydrogen bonding may also play a role as it has been shown to be operative in carbonates and lactones.^38,39^ In contrast, when the first excursion is limited to *ΔV* = 0.8 *V*, the system does not exhibit the same behavior as the applied field is insufficient to trigger a robust dipolar alignment at the interface and the disordered solvent configuration suppresses the emergence of nonlinearity during the hold phase. Here, the poorly oriented dipoles act as a screening layer, attenuating the local electric field and impeding solvent reorganization into the densely packed layer that has been demonstrated to contribute to the overall conductance of the junction. This interpretation is consistent with previous studies on nonlinear dielectric responses, where insufficient field strength prevents dipolar saturation and limits field-induced cooperative effects.^40^ These findings support the established picture of nonlinear dielectric behavior in highly polar liquids under strong electric fields. High-field conditions induce deviations from linear dipolar alignment, leading to complex orientational dynamics and field-dependent dielectric responses.^40^ Electrorheological effects can also emerge, where field-mediated dipole–dipole interactions alter local viscosity and slow down rotational and translational dynamics.^41,42^ Molecular simulations support the idea that strong electric fields promote enhanced dipolar ordering and formation of partially structured interfacial regions.^43^ Under sufficiently high fields, cooperative effects may culminate in electro-freezing-like behavior, where dipolar motion becomes strongly constrained.^37^ These observations demonstrate that the nonlinear response is not only field-dependent but also conditioned by the “initialisation” of the electrode–solvent interface. The first high-field excursion effectively sets the interfacial state, determining whether the system evolves into a highly conductive, ordered regime or remains trapped in a lower-conductivity, weakly correlated configuration. We emphasize that this is one of *several* possible explanations: reorganization of the Au contact under the applied field, changes in the molecule–electrode geometry, local heating, and residual ionic impurities could each contribute, and our measurements do not discriminate among them. As these solvents – particularly propylene carbonate – are widely employed in molecular electronics, their dynamic and nonlinear behavior must be carefully considered when operating beyond steady-state (DC) conditions, with direct implications for the reproducibility of current–voltage characteristics.

**Figure 4:**
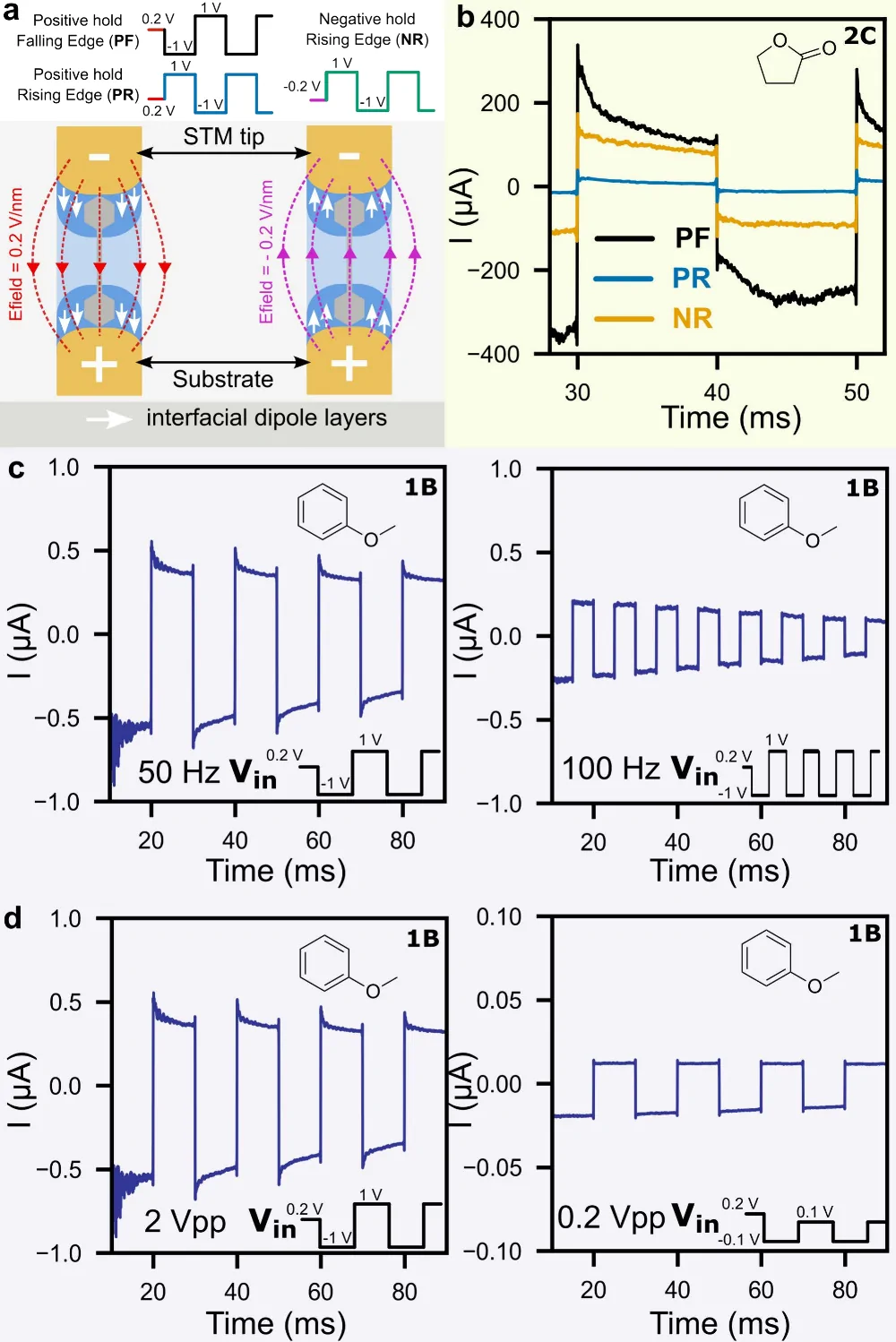
(a) Sketch of the initial-condition tests used to probe the origin of the nonlinear response seen in **Group C** solvents. We set the interfacial dipole layers (white arrows) into two opposite starting orientations by imposing an initial field of +0.2 *V*/*nm* (left) or −0.2 *V*/*nm* (right) across the tip–substrate gap, then drive the junction with the square-wave excitation sketched above each electrode pair. (b) Initial-state dependence measured in γ-butyrolactone, which rules out electrode asymmetry as the source of the dominant nonlinearity. The magnitude of the nonlinear response follows the excursion of the first driving edge: a larger rising or falling edge yields both a stronger nonlinearity and a larger current integrated over the full excitation period. (c, d) Frequency-dependence test in anisole, showing transition from a **Group B** charge/discharge behavior at 50 *Hz* (c) to a near-square trace typical of **Group A** solvents at 100 *Hz* (d). (e, f) Amplitude-dependence test in anisole, isolating the role of field strength in the interfacial reorganization. Reducing amplitude from 2 *V*_*pp*_ (e) to 0.2 *V*_*pp*_ (f) suppresses the transient response (note the rescaled current axis: the modulation falls to a few tens of *nA* and simply follows the bias), which confirms that the charging/discharging of the interfacial layers depends on the applied field strength.

We performed frequency- and amplitude-dependent experiments in anisole (Figure 4c-f) to verify that the behavior observed in **Group B** is also dominated by the response of the solvent itself. Upon doubling the driving frequency from 50 *Hz* to 100 *Hz* (Figure 4c,d), the charging and discharging features associated with the interfacial layers are suppressed, and the response approaches that observed for **Group A** solvents. This behavior suggests the presence of a finite dipolar reorientation time at the electrode surfaces. When the field modulation becomes too rapid, the solvent cannot follow and the system is driven back toward a resistive regime. The amplitude dependence of the response provides additional insight. As shown in Figure 4e,f, reducing the AC bias amplitude by 1 order of magnitude (from 2 *V*_*pp*_ to 0.2 *V*_*pp*_) markedly suppresses charging and discharging features.

In conclusion, we demonstrate that the unique sensitivity of single-molecule junctions (SMJs) to the local electrostatic environment enables their use as high-resolution probes of the inner Helmholtz plane (IHP). By systematically investigating a range of solvents, we identify three distinct behavioral macro-categories: (A) nonpolar solvents, which respond by perfectly following the applied bias, (B) solvents of intermediate polarity, which exhibit a predominantly capacitive response, and (C) highly polar solvents, which strongly deviate from linearity and display pronounced asymmetry with respect to the applied bias. For Groups A and B, the observed behavior correlates closely with macroscopic solvent properties, and the frequency and amplitude dependence of the transients supports a mechanism dominated by field-driven dipolar reorientation. The magnitude of the interfacial follows the driving force for charge transfer between solvent and electrode rather than the molecular dipole moment. In contrast, the response of highly polar solvents cannot be captured within a simple linear or quasi-linear dielectric framework. Instead, it reflects a complex interplay of processes occurring over multiple time scales and driven by the extreme local electric fields (∼1 *GV m* ^–1^) present at the junction. Within this regime, our observations are consistent with the onset of strongly nonlinear dielectric behavior, including field-induced slowing of dipolar dynamics and the emergence of cooperative orientational ordering. The history dependence, phase shifts, and amplitude attenuation observed experimentally point toward interfacial configurations that retain memory of the applied field, in qualitative agreement with mechanisms described in the literature as electro-freezing. We cannot exclude a role for trace impurities (e.g., water) in highly polar solvents. Karl–Fischer titration of the two most hygroscopic solvents in the series places an upper bound on their residual water content (see SI for details), but we draw no further inference from those two measurements. These findings establish SMJs not only as sensitive probes of interfacial electrostatics, but also as a powerful platform to access nonequilibrium and nonlinear dielectric phenomena at the nanoscale. Further work is required to develop a quantitative microscopic description of the mechanisms underpinning the behavior of highly polar solvents such as propylene carbonate and the lactones investigated here, particularly in relation to field-induced ordering and electro-freezing-like transitions at the interface. Having established the “solvent baseline”, we will extend this approach to study electrolytic environments and directly investigate fully developed electric double layers under dynamic conditions and within the domain of frequency.

## Supplementary Material



## Data Availability

All raw data acquired in Liverpool and the code used for its analysis can be accessed free of charge on the University of Liverpool Data Catalogue (deposit no. 3083, DOI:10.17638/datacat.liverpool.ac.uk/3083). The complete postprocessing chain used to extract the equivalent-circuit parameters from the raw traces, including the code that generates Figure 3, is available at https://github.com/Molecular-Electronics-Liverpool/NanoEDL.

## Notes

Additional experimental details, methods employed, materials and experimental conditions, additional data, and supporting calculations (PDF)
