# Supplementary material for: Probing Solvent Dynamics at the Single-Molecule Limit
Source: Nano Lett. 2026 Sep 22;26(39):13415–22. doi: 10.1021/acs.nanolett.6c03272 (PMC13651600; doi:10.1021/acs.nanolett.6c03272)
Supplement: Supplementary file 1 [file nl6c03272_si_001.pdf]

Supporting Information for: Probing Solvent Dynamics at the Single-Molecule Limit

Roberto Listo†[1,2,3], James M. F. Morris†[1], Amit Sil[1], Xiaohang Qiao[1], R. Tom Abram[1], Chanyuan Huo[1,4], Jonathan S. Ward[1,5], Walther Schwarzacher[4,6], Piero Malcovati[2], Marco Vacca[3], Mariagrazia Graziano[7], Giovanna Turvani[3], Richard J. Nichols[1], and Andrea Vezzoli*[1]

[1] Donnan Laboratories, Department of Chemistry, University of Liverpool, Crown Street, Liverpool L69 7ZD, United Kingdom

[2] Department of Electrical, Computer and Biomedical Engineering, University of Pavia, Pavia 27100, Italy

[3] Department of Electronics and Telecommunications, Politecnico di Torino, Torino 10129, Italy

[4] H.H. Wills Physics Laboratory, University of Bristol, Tyndall Avenue, Bristol BS8 1TL, United Kingdom

[5] Department of Chemistry, Lancaster University, Lancaster LA1 4YB, United Kingdom

[6] Syed Babar Ali School of Science and Engineering, Lahore University of Management Sciences, 54792 Lahore, Pakistan

[7] Department of Applied Science and Technology, Politecnico di Torino, Torino 10129, Italy

*Corresponding author: andrea.vezzoli@liverpool.ac.uk
†These authors contributed equally to this work.

Contents

S1 Synthesis 3

S2 Details on the solvent environments 3

S2.1 Solvent screening 4

S2.2 Chlorinated solvents treatment 6

S2.3 Karl–Fischer titration 6

S2.4 Electronegativity as an ordering variable 7

S2.4.1 Calculation of IP and EA 7

S2.4.2 The two scales compared 8

S3 Details on AC-STMBJ data acquisition and processing 9

S3.1 System bandwidth 11

S3.1.1 Input signal and data acquisition 12

S3.1.2 Selection and processing of the traces 13

S3.2 Tips and Substrates 13

S3.3 AC-STMBJ raw conductance vs time data 14

S4 Electrical model and fitting strategy 16

S4.1 Summary of Selected Traces and Fitted Parameters 19

S4.2 Choice of the equivalent circuit: identifiability and model selection 20

S4.2.1 The observable and the parameter-to-observable map 20

S4.2.2 The Randles topology is structurally non-identifiable 21

S4.2.3 Detection limits for the identifiable alternatives 22

S4.2.4 Model selection on the experimental transients 24

S4.2.5 Why no linear circuit can describe Group C 24

S4.3 Re-extraction of the circuit parameters 25

S5 Absence of a mains contribution at $50\,$Hz 26

S5.1 Orthogonality of the mains to the excitation 27

S5.2 Rejection of an incoherent component by the median 27

S5.3 Further controls 29

S6 Validation of the extraction against a network of known composition 29

S1 Synthesis

The molecular wire was synthesised following the procedure previously reported in the literature. The detailed experimental protocols, purification methods, and characterisation data are fully described in the original publication. [1] No modifications to the reported synthetic methodology were introduced.

S2 Details on the solvent environments

Table S1: Static relative permittivity (ε_r), molecular dipole moment (μ), dynamic viscosity (η) and density (ρ) of the solvents used in this work, each value with its source. Most are taken from the *CRC Handbook of Chemistry and Physics*, 97th edition. [2] Where the CRC tables do not list the compound, another source is cited instead. Highlighted rows are new or revised in this revision.

Solvent	ε_r	μ (D)	η (mPa s)	ρ (g mL^{-1})
Mesitylene (1A)	2.279 [2]	0 [2]	0.661 [3]	0.8615 [2]
n-Decane (2A)	1.9853 [2]	0 [2]	0.838 [2]	0.7266 [2]
Anisole (1B)	4.30 [2]	1.2623(14) [4]	1.056 [2]	0.9940 [2]
1,2,4-Trichlorobenzene (2B)	2.24 [5]	1.26 [5]	1.926 [6]	1.459 [2]
1,2-Dichlorobenzene (3B)	10.12 [2]	2.50(5) [2]	1.324 [2]	1.3059 [2]
γ-Valerolactone (1C)	36.9 [7]	4.71(5) [2]	1.82 [8]	1.0551 [2]
γ-Butyrolactone (2C)	39.0 [2]	4.27(3) [2]	1.72 [9]	1.1296 [2]
Propylene carbonate (3C)	66.14 [2]	4.9 [2]	2.50 [10]	1.2047 [2]

The physical properties of the solvents investigated in this work are summarised in Table S1, together with the source of each value. Most are taken from the *CRC Handbook of Chemistry and Physics*, 97th edition; [2] where a compound is absent from the relevant CRC table, a primary measurement is cited in its place. In the course of this revision we re-checked every entry against the CRC tables and their underlying compilations, and corrected several of them. The most consequential correction concerns 1,2,4-trichlorobenzene, whose dipole moment was previously given as 2.70 D: no dipole moment is tabulated for any trichlorobenzene isomer in the standard compilations, and a value of that magnitude is incompatible with the symmetry of the 1,2,4-substituted ring, in which the three substituent moments sum to the moment of a single substituent. The experimental value now quoted, 1.26 D, [5] is consistent with that argument. The table reports the static relative permittivity (ε_r), dynamic viscosity (η), density (ρ) and molec-

ular dipole moment (μ) of the solvents considered in this study. These three quantities provide a compact and physically meaningful description of the solvent environment, combining macroscopic dielectric response with key molecular-scale properties. The static relative permittivity describes a solvent's ability to screen electrostatic interactions. It therefore plays a central role in determining the magnitude and spatial distribution of electric fields, both in the bulk and at interfaces. In contrast, the dynamic viscosity controls the characteristic time scales of molecular translation and reorientation and is thus directly linked to relaxation processes under time-dependent electric fields. For this reason, ε_r and η are particularly relevant for the interpretation of the dynamical measurements reported in this work. The molecular dipole moment provides a microscopic perspective on the dielectric behaviour of the solvents. Although a high static permittivity is often associated with large permanent dipole moments, this relationship is not straightforward and is strongly influenced by molecular packing and intermolecular correlations. Consequently, the simultaneous consideration of ε_r and μ is useful for rationalising differences in the interfacial response observed among solvents with comparable macroscopic dielectric properties.

S2.1 Solvent screening

Twenty-one solvents were screened for compatibility with the AC-STMBJ experiment. A solvent was retained only if it satisfied all four criteria stated in the main text: sufficient solubilising power for the molecular wire at 100 µM, sufficiently low volatility to maintain a stable concentration over the acquisition, liquid state at 298 K, and formation of identifiable single-molecule junctions. The two dominant failure modes were the absence of an identifiable molecular conductance plateau in the one-dimensional histogram, and insufficient solubility of the molecular wire at the working concentration. Table S2 documents the full campaign.

Table S2: Complete solvent screening campaign. Boiling points are quoted at atmospheric pressure. Solvents retained for the study are identified by their group label as used in the main text.

Solvent	b.p. (°C)	Outcome	Reason for exclusion
Mesitylene	165	Retained (1A)	—
n-Decane	174	Retained (2A)	—
Anisole	154	Retained (1B)	—
1,2,4-Trichlorobenzene	214	Retained (2B)	—
1,2-Dichlorobenzene	180	Retained (3B)	—
γ-Valerolactone	207	Retained (1C)	—
γ-Butyrolactone	204	Retained (2C)	—
Propylene carbonate	242	Retained (3C)	—
1,4-Difluorobenzene	89	Excluded	No molecular plateau
1,2-Dimethoxyethane	85	Excluded	No molecular plateau
Toluene	111	Excluded	No molecular plateau
o-Xylene	144	Excluded	No molecular plateau
Chlorobenzene	131	Excluded	No molecular plateau
1-Chloronaphthalene	259	Excluded	No molecular plateau
Ethyl benzoate	212	Excluded	No molecular plateau
Dimethyl sulfoxide	189	Excluded	No molecular plateau
N,N-Dimethylformamide	153	Excluded	No molecular plateau
Water	100	Excluded	Wire insoluble
Ethylene glycol	197	Excluded	Wire insoluble
1,3-Propanediol	214	Excluded	Wire insoluble
Ethylene carbonate	248	Excluded	Solid at 298 K

Glycerol was not screened separately. Its two closest lower homologues, ethylene glycol and 1,3-propanediol, were, and both failed on solubility grounds; the same limitation applies *a fortiori* to glycerol, whose more extended hydrogen-bonded network makes it a poorer solvent still for an apolar aromatic wire. Independently of solubility, the viscosity of glycerol ($\eta \approx 934$ mPa s at 298 K, roughly three orders of magnitude above the solvents of Groups A and B) would place its dipolar relaxation well outside the window accessible with a 100 ms hold sampled at 40 kHz.

S2.2 Chlorinated solvents treatment

Before the measurements, we subjected the chlorinated solvents to additional purification in order to minimise the presence of trace impurities that could influence the electrochemical behaviour of the single-molecule junctions. We passed 1,2-dichlorobenzene and 1,2,4-trichlorobenzene (TCI UK Ltd.) through anhydrous potassium carbonate (Merck). We adopted this treatment to remove residual acidic (HCl) impurities, which are commonly present in chlorinated aromatic solvents due to their synthesis and storage. Even very small amounts of such species can alter interfacial charging processes, leading to spurious or irreproducible responses under high electric fields. We selected anhydrous potassium carbonate as a mild and effective scavenger for acidic contaminants, without chemical modification of the solvent. After treatment, we filtered the solvents, transferred them into clean, dry glassware, and used them immediately. All other solvents were used as received.

S2.3 Karl–Fischer titration

The water content of the solvents used in this work was quantified by Karl–Fischer (KF) titration prior to their use in the single-molecule junction experiments. In particular, anhydrous propylene carbonate and γ-butyrolactone were analysed to estimate the amount of residual water in the solvent batches used throughout the study. KF titration is based on the quantitative reaction between water and iodine in the presence of sulfur dioxide and a base in an alcoholic medium. The consumption of iodine during the titration is stoichiometrically proportional to the amount of water present in the sample, enabling sensitive determination of trace water in organic solvents. Measurements were carried out using a volumetric Karl–Fischer titrator equipped with a sealed titration cell to minimise contamination from atmospheric moisture. Prior to each measurement the system was allowed to stabilise until a constant background drift was reached. Liquid samples were injected into the titration cell through the septum using a gastight syringe to limit exposure to ambient humidity. The titration endpoint was determined potentiometrically. The measured water content was 75 ppm for anhydrous propylene carbonate and 355 ppm for γ-butyrolactone. These values place an upper bound on adventitious water in the two most hygroscopic solvents of the series. We draw no inference from them about a correlation between water content and the magnitude of the nonlinear response, which the present dataset is not designed to test.

S2.4 Electronegativity as an ordering variable

Panels 3e and 3f of the revised manuscript each carry the equivalent capacitance C_{eq} on the left axis and the time constant τ on the right, and differ only in the abscissa: panel 3e uses the molecular dipole moment, panel 3f the quantity

$$\Delta\chi = \chi_{\text{M}} - \Phi_{\text{Au}}, \qquad \chi_{\text{M}} = \frac{\text{IP} + \text{EA}}{2}, \qquad \eta = \frac{\text{IP} - \text{EA}}{2}, \tag{1}$$

where χ_{M} is the Mulliken, or absolute, electronegativity of the solvent molecule, η its chemical hardness, and $\Phi_{\text{Au}} = 5.31$ eV the work function of Au(111) (polycrystalline gold: 5.1 eV). This section documents how those numbers were obtained and why this descriptor was chosen over the empirical alternative.

The physical content of $\Delta\chi$ is the one relevant to the measurement. When solvent and electrode are brought into contact, charge flows between them until the two electronic chemical potentials equalise; $\mu = -\chi$ is that chemical potential, so $\Delta\chi$ is the driving force for the flow and the interface dipole which develops across the inner Helmholtz plane grows with its magnitude. It is therefore a quantity of the same kind as the potential drop that the equivalent circuit measures, rather than a proxy for it. $\Delta\chi$ is negative for all five solvents, that is, charge flows from solvent to metal in every case.

S2.4.1 Calculation of IP and EA

Ionisation potentials and electron affinities were computed by ΔSCF with ORCA 6.1.1. The geometry of the neutral molecule was optimised at the B3LYP-D4/def2-TZVP level (RIJCOSX, def2/J auxiliary basis, TightSCF, TightOpt), and the energies of the cation and the anion were then evaluated at that same geometry, so that IP and EA are *vertical*. Starting geometries were generated with RDKit (ETKDGv3 followed by MMFF94). The inputs, the outputs and the script that extracts the values are deposited with the rest of the analysis (Section S4.3).

The table below collects the results. The computed ionisation potentials agree with the evaluated experimental values of the NIST Chemistry WebBook to 0.11 eV on average and 0.21 eV at worst, which is the accuracy we would expect of this level of theory and is the only external check available on these numbers.

Electronegativity of the five Group A and Group B solvents on the two scales. $\overline{\chi}_{\text{P}}$ is the atom-weighted mean of the atomic Pauling electronegativities over the molecular formula. χ_{M} is the Mulliken electronegativity of the whole

molecule from the computed vertical IP and EA, η the corresponding chemical hardness, and $\Delta\chi = \chi_M - \Phi_{Au}$ the quantity plotted in Figure 3f. χ_P^{calc} maps χ_M onto the Pauling scale through Mulliken's linear relation $\chi_P = 0.374\,\chi_M + 0.17$, with χ_M in electronvolts. Energies in eV.

Solvent	IP	IP_{exp} [a]	EA	χ_M	η	$\Delta\chi$	χ_P^{calc}
Mesitylene (1A)	8.37	8.40	-1.60	3.39	4.98	-1.93	1.44
n-Decane (2A)	9.81	9.65	-1.95	3.93	5.88	-1.38	1.64
Anisole (1B)	8.18	8.20	-1.59	3.30	4.88	-2.02	1.40
1,2,4-Trichlorobenzene (2B)	8.83	9.04	-0.53	4.15	4.68	-1.16	1.72
1,2-Dichlorobenzene (3B)	8.92	9.06	-0.91	4.01	4.92	-1.30	1.67

[a] Evaluated ionisation energies, NIST Chemistry WebBook.

Atom-weighted mean Pauling electronegativity $\overline{\chi}_P$ of all eight solvents, computed from the molecular formula with $\chi(H) = 2.20$, $\chi(C) = 2.55$, $\chi(O) = 3.44$ and $\chi(Cl) = 3.16$; hydrogen atoms are counted explicitly.

Solvent	Formula	$\overline{\chi}_P$
Mesitylene (1A)	C_9H_{12}	2.350
n-Decane (2A)	$C_{10}H_{22}$	2.309
Anisole (1B)	C_7H_8O	2.431
1,2,4-Trichlorobenzene (2B)	$C_6H_3Cl_3$	2.615
1,2-Dichlorobenzene (3B)	$C_6H_4Cl_2$	2.535
γ-Valerolactone (1C)	$C_5H_8O_2$	2.482
γ-Butyrolactone (2C)	$C_4H_6O_2$	2.523
Propylene carbonate (3C)	$C_4H_6O_3$	2.594

S2.4.2 The two scales compared

We first ordered Group B by $\overline{\chi}_P$, the atom-weighted mean of the atomic Pauling electronegativities, and the manuscript was written around that descriptor before we adopted $\Delta\chi$. The two scales place Group B in the same order — anisole, then 1,2-dichlorobenzene, then 1,2,4-trichlorobenzene — so the trend reported in Figure 3f does not depend on which is used, and C_{eq} and τ fall monotonically along the sequence either way. We report both here for that reason.

There are nonetheless three arguments for the Mulliken quantity, and they are why the figure uses it. First, χ_M is a property of the molecule, obtained from its own IP and EA, whereas $\overline{\chi}_P$ is an average over atoms with a weighting we would have to choose; the atom count is the obvious choice but it is a choice, and nothing in the physics requires it. Second, referencing to Φ_{Au} turns the abscissa into the driving force for interfacial charge transfer, which connects the

chemistry to the electrical model instead of merely correlating with it. Third, the Pauling scale is defined for atoms in molecules and not for whole molecules at all; the last column of the first table below maps χ_M onto it for comparison, and the resulting values, between 1.4 and 1.7, sit far below the atom-weighted means because a molecule is far less electronegative than the atoms composing it. The two columns are not the same quantity and should not be read as alternative estimates of one.

The two scales do differ in one respect. Group A reverses between them: on the Pauling scale decane precedes mesitylene, on the Mulliken scale mesitylene precedes decane. Nothing in the manuscript rests on that ordering, since C_eq is negligible for both, but it illustrates that an atom-weighted mean is not a conserved ranking — carbon is more electronegative than hydrogen on the Pauling scale and less so on the Mulliken one, which is enough to invert two hydrocarbons.

Three limitations should be stated plainly. The electron affinities of these molecules are negative: the anion is a resonance in the continuum rather than a bound state, and the def2-TZVP basis, which carries no diffuse functions, confines it artificially and so returns a finite and reproducible number. This is the usual pragmatic choice when relative values across similar molecules are wanted, but adding diffuse functions would move those numbers. Next, the experimental ionisation potentials of the two chlorinated benzenes are equal within their uncertainties, 9.06 eV and 9.04 eV, so their separation in χ_M comes entirely from the computed electron affinities; that ordering is the least secure link in the chain. Finally, three solvents cannot establish a functional form, and within Group B $\Delta\chi$ ranks them in the same order as the viscosity. The correlation is presented in the manuscript as qualitative for these reasons.

S3 Details on AC-STMBJ data acquisition and processing

We employed our system *LEVIATHAN III*, a modified Keysight 5500 STM, to fabricate and characterise single-molecule junctions. We have described the instrument in detail in our previous publications. [11–13] Recent software & hardware upgrades were described in the supporting information of Morris et al. [14]

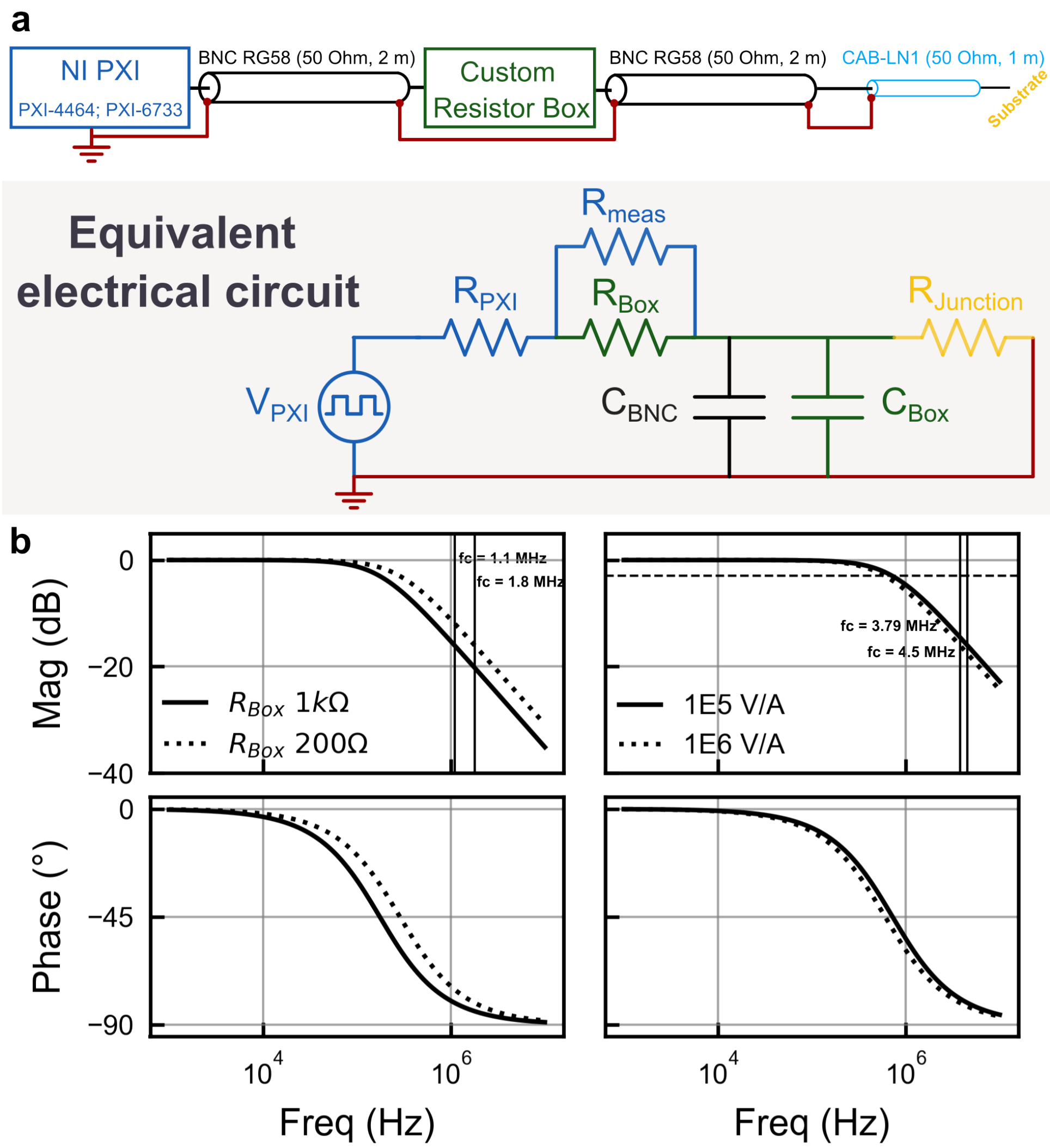

Figure S1: a) Schematic illustrating the AC generation branch of our *LEVIATHAN III* with the associated equivalent electrical circuit for estimating the frequency behaviour of the system. b) Bode diagrams of the equivalent electrical circuit. The left panels illustrate the effect of the external series resistance (R_{Box}) on the voltage attenuation and phase, while the right panels compare the calculated system bandwidth and phase for transimpedance amplifier gains of 10^5 V A^{-1} and 10^6 V A^{-1}.

S3.1 System bandwidth

For AC-STMBJ measurements, we studied the system's parasitics to tune the settings appropriately. Figure S1a shows the block diagram of the measurement setup together with the corresponding equivalent electrical circuit used to estimate the effective bandwidth of the bias line. In this representation, the voltage source is modelled as an ideal generator in series with its output resistance R_{PXI}, followed by the external series resistance R_{Box} introduced by the resistor box. The junction node is connected to ground through an equivalent capacitance C_{BNC}, which accounts for the combined parasitic capacitance of the coaxial cables and connectors, while the capacitive contribution due to the resistor box is represented by a parallel C_{Box}. Because the junction itself presents a very high impedance, its resistive contribution is represented as R_{Junction}. However, the transimpedance amplifier in our setup (Femto DLPCA-200) is configured to hold the source input at $0\,\text{V}$ such that only the DC input impedance (R_{in}) contributes to the bandwidth of the instrument.

The total capacitance can be expressed as

$$C_{\text{tot}} = C_{\text{BNC}} + C_{\text{Box}}, \tag{2}$$

where C_{BNC} is the capacitance of the coaxial cables, and C_{box} the parasitic capacitance associated with the resistor box. The dominant contribution arises from the coaxial cables. For standard RG58 cables, the capacitance per unit length is approximately

$$C' \approx 100\,\text{pF}\,\text{m}^{-1}.$$

Given a total cable length of $L = 5\,\text{m}$, this yields

$$C_{\text{BNC}} = C'L \approx 500\,\text{pF}. \tag{3}$$

The resistor box parasitic contribution was measured to be

$$C_{\text{BOX}} = 200\,\text{pF}.$$

The total capacitance is therefore estimated as

$$C_{\text{tot}} = 700\,\text{pF}.$$

The equivalent circuit behaves as a first-order low-pass filter with a time constant $\tau = R_{\text{in}} C_{\text{tot}}$ and a corresponding cutoff frequency

$$f_{\text{c}} = \frac{1}{2\pi R_{\text{in}} C_{\text{tot}}}. \tag{4}$$

Based on the specifications of the transimpedance amplifier (Femto DLPCA-200), the DC input impedance R_{in} varies with the selected gain setting, which in turn affects both the instrument's bandwidth and the effective voltage at the junction (Figure S1b, right panels). At a gain of $10^5\,\text{V A}^{-1}$, the input impedance is $R_{\text{in}} = 50\,\Omega$, yielding:

$$\tau_{\text{1E5}} \approx 35\,\text{ns} \quad \text{and} \quad f_{\text{c,1E5}} \approx 4.5\,\text{MHz}.$$

At a higher gain of $10^6\,\text{V A}^{-1}$, the input impedance increases slightly to $R_{\text{in}} = 60\,\Omega$, resulting in:

$$\tau_{\text{1E6}} \approx 42\,\text{ns} \quad \text{and} \quad f_{\text{c,1E6}} \approx 3.79\,\text{MHz}.$$

For both the $10^5\,\text{V A}^{-1}$ and $10^6\,\text{V A}^{-1}$ settings, the calculated RC cutoff frequencies are well above the amplifier's internal bandwidth limit of $200\,\text{kHz}$ and $400\,\text{kHz}$ for $10^6\,\text{V A}^{-1}$ and $10^5\,\text{V A}^{-1}$ respectively. This means the system speed is fundamentally constrained by the amplifier itself, and the parasitic capacitance does not limit the low-frequency square-wave excitations used in these experiments.

Furthermore, because the junction itself presents a very high impedance, the applied bias voltage undergoes attenuation across the voltage divider formed by the external series resistor R_{Box} and the amplifier's internal input impedance R_{in}. As illustrated in the left panels of Figure S1b, altering the resistor box value changes this attenuation profile. To minimise excessive voltage drop and distortion of the excitation waveform whilst preserving the junction stability and low-noise environment afforded by the series resistor, we limited R_{Box} to $1\,\text{k}\Omega$.

S3.1.1 Input signal and data acquisition

AC-STMBJ measurements were performed on the same molecule in different environments. For each measurement, the molecule was trapped for $100\,\text{ms}$, while a square-wave bias modulation at $50\,\text{Hz}$ was applied to the junction. This frequency was selected to ensure that the junction conductance could reach a steady state within each half-cycle. Both current and volt-

age signals were simultaneously acquired at a sampling rate of 40 kHz.

Because 50 Hz coincides with the European mains frequency, we verified explicitly that mains pickup does not contribute to the measured transients. Two arguments apply. First, the excitation is generated by the PXI source and is not phase-locked to the mains, so each junction hold samples an independent mains phase and the point-by-point median across the thousands of retained traces rejects any incoherent 50 Hz component as $1/\sqrt{N}$. Second, and more directly, a square wave contains odd harmonics only: in our 100 Hz dataset the excitation occupies 100, 300, 500 Hz and so on, so 50 Hz is orthogonal to the drive over the 100 ms record and any amplitude measured there is mains pickup, free of spectral leakage. That measurement returns 1.6 pA, more than an order of magnitude below the instrumental noise floor and statistically indistinguishable from the background of non-drive spectral bins. The full analysis is given in Section S5.

S3.1.2 Selection and processing of the traces

Only traces corresponding to successfully formed single-molecule junctions were retained for further analysis. The selection procedure was based on the molecular plateau's conductance value. In particular, a Gaussian filter centred on the characteristic conductance of the molecular junction was applied in order to identify traces consistent with the expected conductance signature of the molecule. Traces falling within the selected conductance window were retained, while traces outside this range were discarded. The trace selection was performed using the same data-processing code routinely employed within the group for the analysis of scanning tunnelling microscope break-junction (STMBJ) measurements and previously used in related studies. [15] For each solvent environment, the traces selected through this procedure were grouped into a dataset, and a median trace was calculated. Using the median reduces the influence of outliers and occasional junction instabilities, providing a representative time-dependent response for each solvent system.

S3.2 Tips and Substrates

In this work, we used substrates prepared by e-beam evaporation of 200 nm of Au (99.99+%, Goodfellow Cambridge Ltd) on freshly cleaved muscovite mica (Agar Scientific Ltd).

Table S3 summarise the type of tip used in the measurements made in every solvent environ-

ment. We used Au (99.99+%, Goodfellow Cambridge Ltd) tips insulated with wax (Apiezon Wax W40, M&I Materials Ltd) or PVDF (3D Xtech PVD1010750N FluorX™ PVDF Filament).

Table S3: Type of STM tip used for measurements performed in each solvent environment.

Solvent	Tip material	Tip treatment
Mesitylene	Au	Uncoated
n-Decane	Au	Uncoated
Anisole	Au	Uncoated
1,2,4-Trichlorobenzene	Au	Uncoated
1,2-Dichlorobenzene	Au + PVDF	Coated
γ-Valerolactone	Au + WAX	Coated
γ-Butyrolactone	Au + WAX	Coated
Propylene carbonate	Au + WAX	Coated

S3.3 AC-STMBJ raw conductance vs time data

To provide a direct overview of the time-dependent conductance response before the extraction of the equivalent electrical parameters, the selected AC-STMBJ traces were represented as two-dimensional conductance histograms. For each dataset, the retained traces were aligned on the waveform period and binned as a function of time and log(G/G_0) over the central portion of the acquisition window, from 10 to 90 ms. This representation allows the full conductance distribution to be visualised, rather than only the median profile used in the subsequent post-processing analysis.

The white curve overlaid on each histogram corresponds to the median log(G/G_0) profile calculated point-by-point across all retained traces. This is the same median conductance profile used as input for the following post-processing steps, including detrending, time-window segmentation, and equivalent circuit parameter extraction. The histograms therefore provide a direct visual check of the relationship between the raw retained trace distribution and the representative median signal used for the quantitative analysis.

The number of traces contributing to each distribution is reported in Table S4. The different hit rates reflect the dataset-dependent fraction of traces satisfying the selection criteria and should therefore be considered when comparing the apparent sharpness and density of the

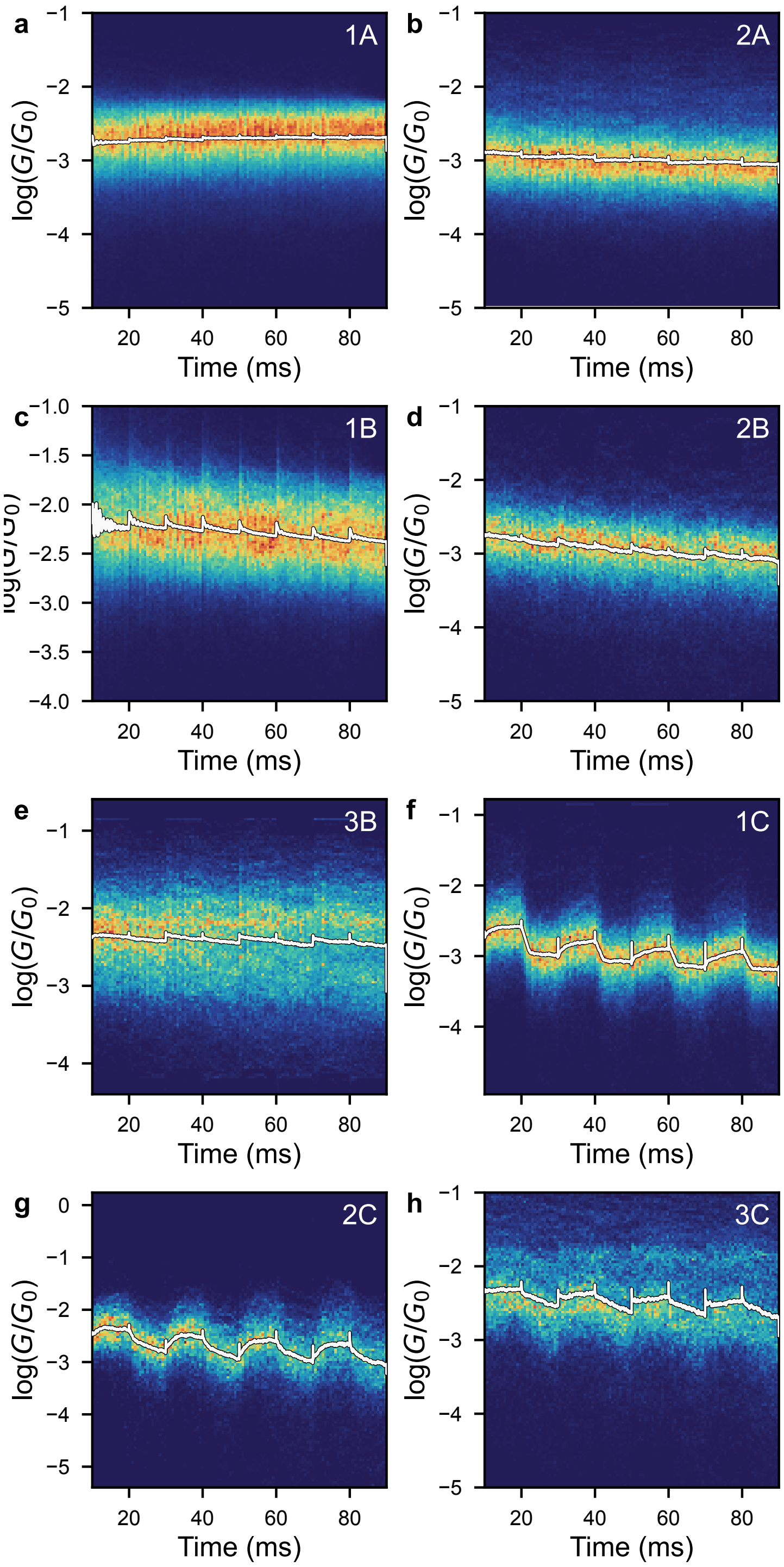

Figure S2: Two-dimensional histograms of the retained AC-STMBJ conductance traces as a function of time for the analysed solvent datasets. Each panel shows the binned distribution of $\log(G/G_0)$ values between 10 and 90 ms, after the trace selection procedure. The white line overlaid on each histogram represents the point-by-point median $\log(G/G_0)$ profile across the retained traces, corresponding to the representative profile used in the subsequent post-processing workflow. Panels solvent's environments: (a) 1A, (b) 2A, (c) 1B, (d) 2B, (e) 3B, (f) 1C, (g) 2C, and (h) 3C.

corresponding two-dimensional histograms.

Table S4: Summary of the number of traces retained after the data selection procedure for each dataset. The table reports the initial number of raw traces, the number of valid traces retained after processing, and the corresponding hit rate percentage.

Dataset	Initial traces	Retained traces	Hit rate (%)
Mesitylene	11665	8936	76.60
Anisole	9724	5081	52.25
n-Decane	8475	4345	51.27
1,2-DCB	9766	2951	30.22
GVL	3167	1736	54.81
GBL	6374	884	13.87
PC	8813	2162	24.53
1,2,4-TCB	3400	1362	40.06

S4 Electrical model and fitting strategy

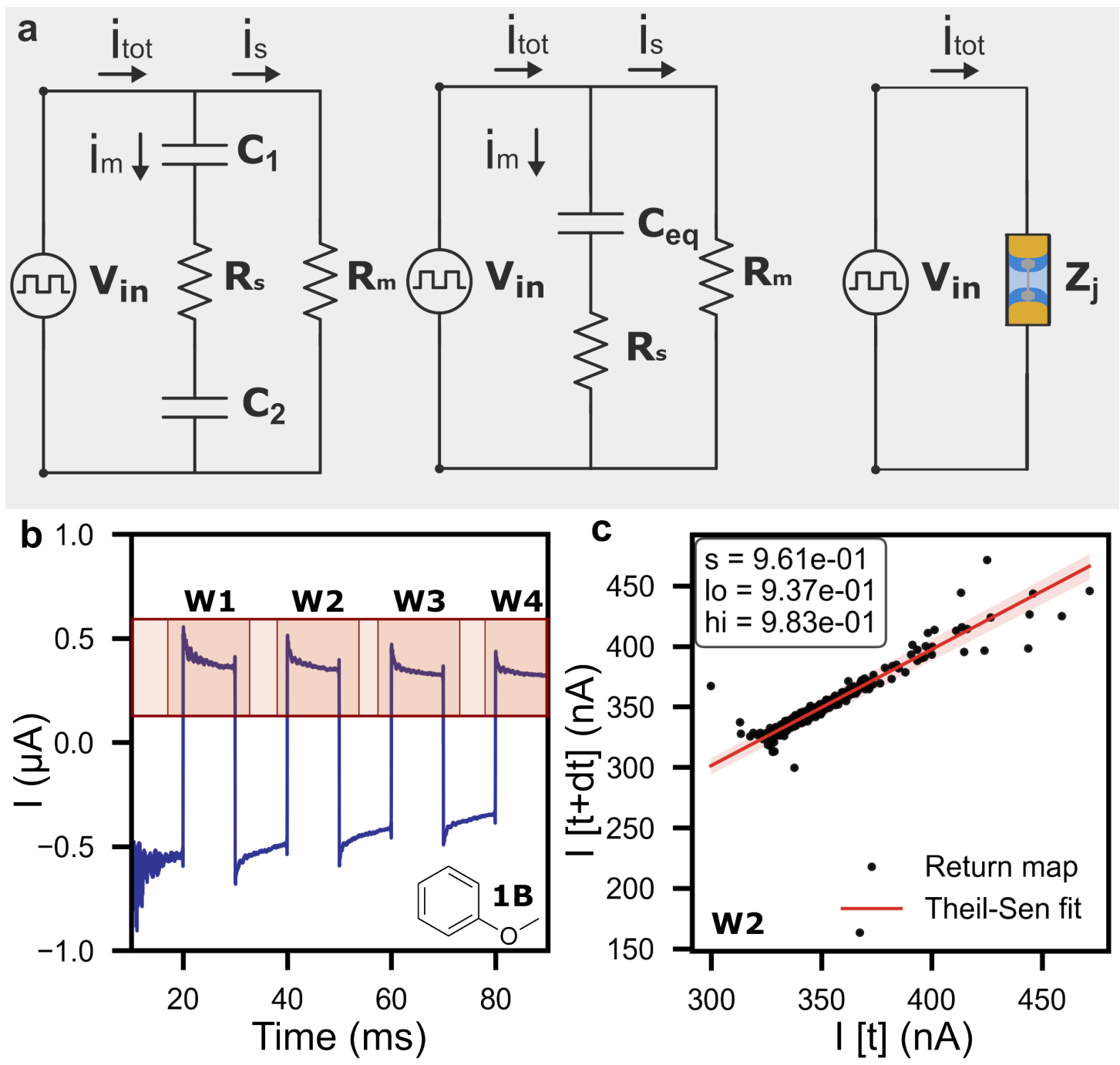

Figure S3: (a) Equivalent electrical circuit of the single-molecule junction environment under study. (b) Representative positive windows selected for the fitting procedure for the current response in anisole. (c) Theil–Sen estimator applied to the return-map scatter plot corresponding to the second current window (W2) in anisole.

The measured current is described using the equivalent circuit shown in Figure S3a, which comprises two parallel contributions: a resistance R_m, accounting for the single-molecule junction, and a series C_1–R_s–C_2 branch representing the electric double layer (EDL). We simplified the electrode interface of the EDL branch by removing the parallel charge transfer resistors R_{ct}

used in electrochemical equivalent circuit models. This is because no Faradaic currents were observed above our instrument noise floor due to either low solvent polarity or, in the case of more polar solvents, coating the STM tip to reduce the Faradaic currents to unobservable levels. Thus, the R_{ct} sub-branch provides insignificant current contributions, which simplifies and improves the model fitting. The physical significance of R_s is described in the main text. The system is driven by a square-wave voltage $v(t) = \pm V_0$ with frequency f and half-period $\Delta = 1/(2f)$. The two capacitors are combined into an equivalent capacitance

$$C_{\text{eq}} = \frac{C_1 C_2}{C_1 + C_2},$$

which defines the characteristic time constant of the EDL branch:

$$\tau = R_s C_{\text{eq}}.$$

The total current can be written as

$$i(t) = i_m(t) + i_s(t),$$

where the molecular contribution is an amplitude $B = (V_0^+ - V_0^-)/R_m$, and the EDL contribution consists of an exponential transient following each voltage step with amplitude $A = (V_0^+ - V_0^-)/R_s$. Within each half-cycle, the current is therefore expressed as

$$i(t) = A\, e^{-\frac{t-t_0}{\tau}} + B,$$

where t_0 denotes the switching time.

The fitting procedure exploits the piecewise-constant nature of the excitation. As shown in Figure S3b, the current trace is divided into time windows (W1–W4 in anisole), each corresponding to a half-cycle following a voltage transition. Since the applied voltage remains constant within each window, the EDL response reduces to a single-exponential relaxation, while the molecular contribution provides a constant offset. Each window is therefore fitted independently, allowing for cycle-to-cycle variations without enforcing periodicity.

To robustly extract the relaxation time, the exponential model is reformulated in discrete time.

Starting from

$$i(t) = A\, e^{-\frac{t-t_0}{\tau}} + B,$$

the current at time $t + dt$ can be written as

$$i(t+dt) = A\, e^{-\frac{t-t_0}{\tau}} e^{-\frac{dt}{\tau}} + B.$$

Using $i(t) - B = A\, e^{-\frac{t-t_0}{\tau}}$, this becomes

$$i(t+dt) = e^{-\frac{dt}{\tau}}\, i(t) + B\left(1 - e^{-\frac{dt}{\tau}}\right).$$

This expression defines a linear relation of the form

$$y = nx + c,$$

with

$$y = i(t+dt), \qquad x = i(t),$$

$$n = e^{-\frac{dt}{\tau}}, \qquad c = B\left(1 - e^{-\frac{dt}{\tau}}\right).$$

Accordingly, the transient current within each window can be represented through a return-map plot, where $i(t+dt)$ is plotted as a function of $i(t)$. An example is shown in Figure S3c for the second window (W2) in anisole. In this representation, the data follow a linear trend, and the parameters n (slope) and c (intercept) are extracted using a Theil–Sen estimator [16], providing robustness against noise and outliers. A similar strategy has been employed in the analysis of transient currents in single-molecule junctions (see Supporting Information of our previous work [17]).

Once n and c are determined, the physical parameters are recovered as

$$\tau = -\frac{dt}{\ln n}, \qquad B = \frac{c}{1-n}.$$

The amplitude A is then obtained by fitting the original current trace within each window using the exponential model with B and τ fixed.

The fitted parameters are mapped onto the circuit elements as follows. The molecular resis-

tance is obtained from the steady-state current,

$$R_m = \frac{(V_0^+ - V_0^-)}{B},$$

while the time constant provides the product

$$\tau = R_s C_{\text{eq}}.$$

The series resistance is determined from the transient amplitude,

$$R_s = \frac{(V_0^+ - V_0^-)}{A},$$

and the equivalent capacitance follows as

$$C_{\text{eq}} = \frac{\tau}{R_s}.$$

We note that only the product $R_s C_{\text{eq}}$ is directly constrained by the dynamics; therefore, the extracted values should be interpreted as effective parameters describing the EDL response.

S4.1 Summary of Selected Traces and Fitted Parameters

To ensure full transparency and reproducibility of our analysis, all fitted parameters extracted from the experimental data are summarised in Table S5. The table reports the series resistance (R_s), time constant (τ), equivalent capacitance (C_{eq}) and molecular resistance (R_m) obtained for each solvent dataset. Values are averages over the individual half-periods of the square-wave excitation, quoted as mean $\pm$ SEM.

The parameters reported here were re-extracted during this revision, following the reviewers' queries on the magnitude of C_{eq} and on the reliability of τ. Three changes were made to the extraction, all documented in Section S4.3: the voltage used to convert the fitted amplitudes into resistances is now measured half-period by half-period rather than assumed; the first and last segments of each acquisition, which are held at the DC set-point bias rather than driven by the square wave, are excluded; and C_{eq} is obtained as τ/R_s throughout. The values below supersede those of the original submission. The principal effect is on R_s, which is now referred to the full $2\,\text{V}$ step at the switching edge and is therefore twice its previous value, and

correspondingly on C_{eq}, which is halved. All Group C solvents are now included.

Table S5: Summary of fitted parameters obtained for each solvent dataset. Values are reported as mean $\pm$ SEM across the half-periods retained for analysis (n given in the last column).

Solvent	R_s (MΩ)	τ (ms)	C_{eq} (pF)	R_m (MΩ)	n
Mesitylene (1A)	471 ± 174	0.098 ± 0.022	0.7 ± 0.4	6.57 ± 0.13	8
n-Decane (2A)	386 ± 54	0.081 ± 0.026	0.3 ± 0.2	12.56 ± 0.54	8
Anisole (1B)	17.0 ± 1.3	0.895 ± 0.136	54.1 ± 8.7	2.62 ± 0.17	6
1,2,4-TCB (2B)	84.8 ± 8.5	0.276 ± 0.033	3.6 ± 0.6	11.64 ± 0.96	8
1,2-DCB (3B)	44.0 ± 11.1	0.319 ± 0.039	9.3 ± 1.6	3.36 ± 0.10	8
γ-Valerolactone (1C)	147 ± 65	0.629 ± 0.193	38.7 ± 14.8	4.10 ± 0.38	8
γ-Butyrolactone (2C)	14.4 ± 1.4	0.766 ± 0.146	60.1 ± 15.3	8.01 ± 1.44	8
Propylene carbonate (3C)	33.0 ± 7.1	0.633 ± 0.127	21.9 ± 3.8	12.92 ± 1.87	8

For anisole the first complete cycle of the square wave was excluded in addition to the DC segments, leaving six half-periods: the excitation there is still settling from the set-point bias, and the time constant returned by that first cycle differs from the remainder by more than an order of magnitude. The Group C entries are reported for completeness; as Section S4.2.5 shows, the response of those solvents is not linear, so the parameters quoted for them describe the fitted waveform rather than a physically meaningful equivalent circuit.

S4.2 Choice of the equivalent circuit: identifiability and model selection

The circuit of Figure S3a was adopted after evaluating three alternative topologies, shown in Figure S4a–d and implemented as the SPICE netlist deposited with the data. This section documents that comparison. All waveforms are the periodic steady-state response of the network to the square-wave drive, synthesised in the frequency domain and rolled off by the amplifier bandwidth.

Throughout we take the instrumental noise floor to be $\sigma_I \approx 58$ pA, obtained from the input-referred current noise density of the DLPCA-200 ($\sim 1.3 \times 10^{-13}$ A Hz$^{-1/2}$) over its 200 kHz bandwidth.

S4.2.1 The observable and the parameter-to-observable map

The EDL branch of the adopted circuit has impedance

$$Z_{\text{b}}(s) \;=\; R_{\text{s}} + \frac{1}{s\,C_{\text{eq}}}, \qquad \frac{1}{C_{\text{eq}}} \;=\; \frac{1}{C_1} + \frac{1}{C_2}, \tag{5}$$

so that the response to a voltage step of amplitude ΔV at $t = t_0$ is the three-parameter waveform

$$i(t) \;=\; A\,e^{-(t-t_0)/\tau} + B, \qquad A = \frac{\Delta V}{R_\mathsf{s}}, \quad B = \frac{\Delta V}{R_\mathsf{m}}, \quad \tau = R_\mathsf{s} C_\mathsf{eq}. \tag{6}$$

The parameter-to-observable map $\Phi_0 : (R_\mathsf{m}, R_\mathsf{s}, C_\mathsf{eq}) \mapsto (A, B, \tau)$ defined by Equation 6 is a bijection: the adopted circuit is exactly identifiable, carrying as many free parameters as the waveform carries independent quantities. This is the benchmark against which the alternatives must be judged.

Equation 5 already contains the first result. The two interfacial capacitances enter only through their series combination, so the level set $\{(C_1, C_2) : C_1 C_2/(C_1+C_2) = C_\mathsf{eq}\}$ is a one-dimensional family of parameter pairs producing identical currents. Retaining C_1 and C_2 as independent parameters adds a degree of freedom to which the experiment is blind: numerically, taking $C_1/C_2 = 100$ at fixed C_eq changes the predicted current by RSS$/\sigma_I^2 \approx 5 \times 10^{-20}$, against a detection threshold of 3.84. C_eq is therefore the maximal identifiable description of the interfacial capacitance.

S4.2.2 The Randles topology is structurally non-identifiable

Shunting each interfacial capacitor with a charge-transfer resistance R_ct (Figure S4b) gives the branch impedance

$$Z_\mathsf{b}^{(1)}(s) \;=\; R_\mathsf{s} + \frac{2R_\mathsf{ct}}{1 + s\,R_\mathsf{ct} C}, \qquad C = 2C_\mathsf{eq}, \tag{7}$$

which still has a single pole. The step response therefore retains the form of Equation 6, with

$$B_1 = \Delta V\left(\frac{1}{R_\mathsf{m}} + \frac{1}{R_\mathsf{s} + 2R_\mathsf{ct}}\right), \quad A_1 = \Delta V\left(\frac{1}{R_\mathsf{s}} - \frac{1}{R_\mathsf{s} + 2R_\mathsf{ct}}\right), \quad \tau_1 = \frac{R_\mathsf{s} R_\mathsf{ct} C}{R_\mathsf{s} + 2R_\mathsf{ct}}. \tag{8}$$

The map $\Phi_1 : (R_\mathsf{m}, R_\mathsf{s}, C_\mathsf{eq}, R_\mathsf{ct}) \mapsto (A_1, B_1, \tau_1)$ sends four parameters to three observables, so its fibres are one-dimensional and R_ct cannot be recovered. The degeneracy is explicit: the Randles circuit with parameters $(R_\mathsf{m}, R_\mathsf{s}, C_\mathsf{eq}, R_\mathsf{ct})$ produces exactly the current of the adopted circuit with

$$\frac{1}{R_\mathsf{m}^\star} = \frac{1}{R_\mathsf{m}} + \frac{1}{R_\mathsf{s} + 2R_\mathsf{ct}}, \qquad \frac{1}{R_\mathsf{s}^\star} = \frac{1}{R_\mathsf{s}} - \frac{1}{R_\mathsf{s} + 2R_\mathsf{ct}}, \qquad C_\mathsf{eq}^\star = \frac{\tau_1}{R_\mathsf{s}^\star}. \tag{9}$$

Equation 9 shows that R_{ct} introduces no new physics into the observable: it merely re-partitions the DC conductance between the molecular and the EDL branch. In the limit $R_{\text{ct}} \gg R_{\text{s}}$ the starred parameters revert to the unstarred ones, as they must.

Figure S4e,f confirms this numerically. Sweeping R_{ct} over four decades and re-fitting the remaining parameters reproduces the adopted response to an RMS difference below 4×10^{-22} A, about 10^{-12} of σ_I; the only visible consequence is the drift of the fitted R_{m}, which for anisole moves from 2.50 to $3.15\,\text{M}\Omega$ in the extreme case $R_{\text{ct}} = R_{\text{s}}/2$ and by less than $0.2\,\%$ once $R_{\text{ct}} \geq 100\,R_{\text{s}}$. Faradaic currents are below the noise floor in every dataset, either because the solvent is of sufficiently low polarity (Group A, together with anisole and 1,2,4-trichlorobenzene, all measured with uncoated tips) or because the tip is coated (PVDF for 1,2-dichlorobenzene, wax throughout Group C; Table S3), so the latter regime applies and the bias from omitting R_{ct} is negligible.

S4.2.3 Detection limits for the identifiable alternatives

Two alternatives do add genuine degrees of freedom: a second, slower interfacial relaxation carrying a fraction ϕ of the interfacial capacitance (Figure S4c), and a constant-phase element of exponent α (Figure S4d), with impedance

$$Z_{\text{CPE}}(s) = \frac{1}{Q\,s^{\alpha}}, \qquad 0 < \alpha \leq 1, \tag{10}$$

which reduces to an ideal capacitor for $\alpha = 1$. Both contain the adopted circuit as a nested special case, so the appropriate test is the likelihood ratio

$$\Lambda = \frac{1}{\sigma_I^2} \sum_{k=1}^{N} \big[\, i_{\text{alt}}(t_k) - i_{\text{M0}}(t_k; \hat{\theta}) \,\big]^2 \; > \; \chi^2_{1,\,0.95} = 3.841, \tag{11}$$

where $\hat{\theta}$ are the adopted-circuit parameters that best reproduce the alternative and $N = 4000$ is the number of samples in a junction hold. At the instrumental noise floor a second relaxation five times slower than the main one becomes resolvable only once it carries more than $7.8\,\%$ of the interfacial capacitance ($12.5\,\%$ at a conservative $300\,$pA), whereas the CPE exponent is constrained to $1 - \alpha \lesssim 6 \times 10^{-5}$. That bound is far tighter than any physically meaningful dispersion, which means a statistically significant $\alpha \neq 1$ would report residual baseline drift rather than interfacial physics — and is precisely why the traces are detrended before fitting.

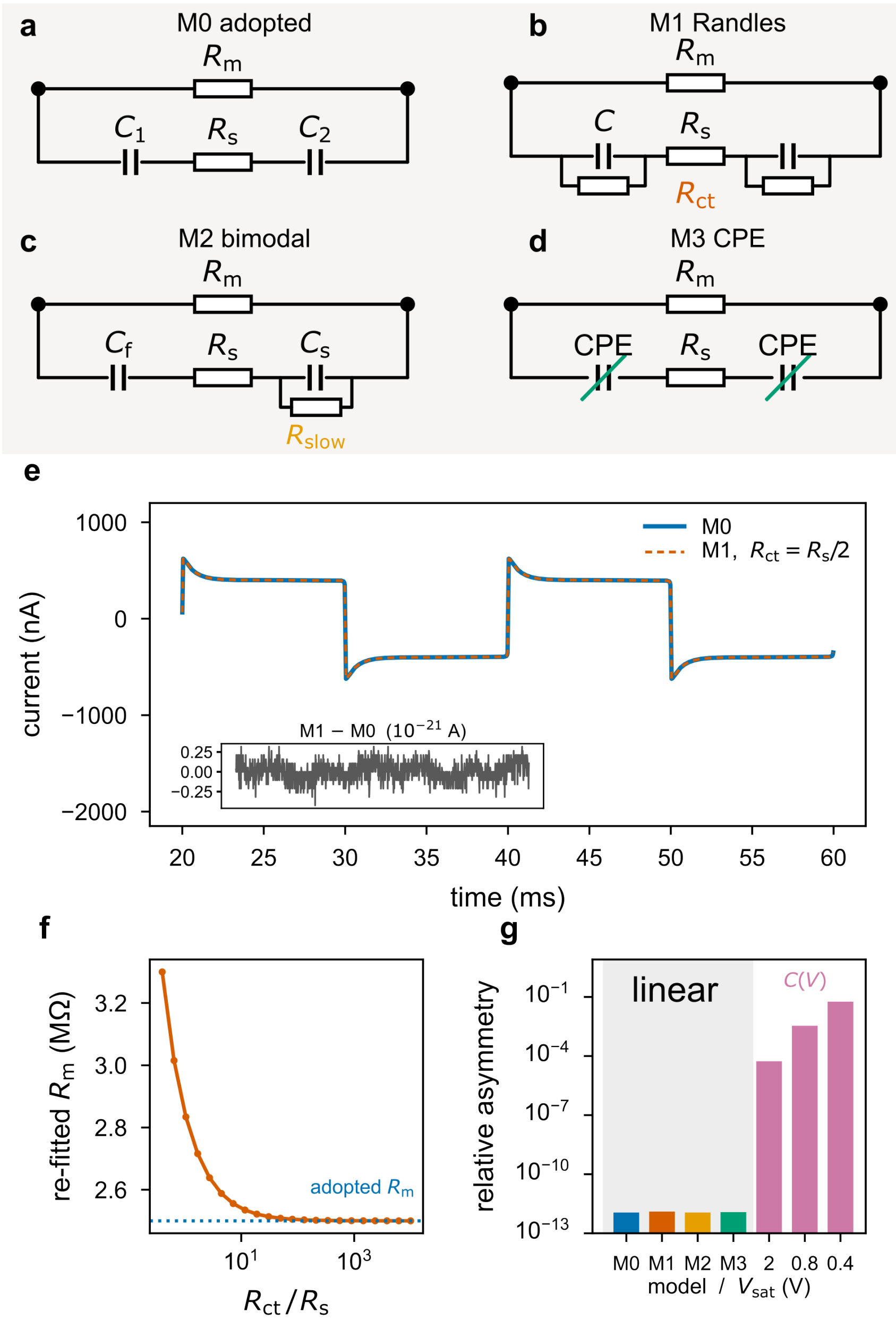

Figure S4: Candidate equivalent circuits and the Randles degeneracy. (a–d) The four topologies compared, as implemented in the deposited SPICE netlist. (e) Periodic steady-state current of the adopted circuit (anisole parameters) and of the Randles circuit with $R_{ct} = R_s/2$ after re-fitting; the inset shows their difference, which stays at the level of 1×10^{-21} A. (f) Re-fitted Rm against Rct/Rs, illustrating the conductance re-partitioning; the inset shows the RMS residual normalised by the instrumental noise floor. (g) Relative half-wave asymmetry: every linear topology sits at machine precision whereas a field-dependent capacitance generates asymmetry that grows as the saturation voltage enters the experimental bias range.

S4.2.4 Model selection on the experimental transients

Each topology was fitted to the experimental median transient over the 10–90 ms analysis window, driven by the *measured* median voltage waveform rather than an idealised square wave, so that phase, amplitude, duty cycle and any residual distortion of the excitation are taken from the experiment: $I_{\text{model}}(f) = V_{\text{meas}}(f)\,Y_{\text{model}}(f)$, following the standard treatment of dynamical systems driven by non-ideal inputs. [18] Models were ranked by

$$\text{BIC} \;=\; N\ln\!\left(\frac{\text{RSS}}{N}\right) + k\ln N, \tag{12}$$

with k the number of free parameters. Table S6 reports the outcome. The adopted three-parameter circuit is preferred in every solvent for which a transient is present, and the Randles and bimodal variants return residuals numerically identical to it — the empirical counterpart of the degeneracy of Equation 9.

Table S6: Model comparison on the experimental median transients. M0: adopted circuit; M1: Randles; M2: bimodal relaxation; M3: constant-phase element. A positive ΔBIC favours M0.

Solvent	Traces	RMSE/span (M0)	ΔBIC (M1)	ΔBIC (M2)	ΔBIC (M3)
Mesitylene	2000	3.2%	$+8.6$	$+9.4$	$+21.7$
n-Decane	2000	4.5%	-14.5	$+8.1$	-3.2
Anisole	2000	3.2%	$+8.1$	$+8.1$	$+21.0$
1,2-DCB	2000	6.2%	$+8.1$	$+8.1$	$+19.3$
1,2,4-TCB	1362	7.3%	$+8.1$	$+8.1$	$+23.6$

In *n*-decane alone the Randles variant is marginally preferred, but decane is a Group A solvent whose fitted capacitance is 0.1 pF: there is no transient to model, and the comparison is between two descriptions of a featureless response.

S4.2.5 Why no linear circuit can describe Group C

Let the network be linear and time-invariant with impulse response h, so that $i(t) = \int_0^\infty h(u)\,v(t-u)\,\mathrm{d}u$. If the excitation is half-wave antisymmetric, $v(t+T/2) = -v(t)$ for all t, then in periodic steady state

$$i\big(t+\tfrac{T}{2}\big) = \int_0^\infty h(u)\,v\big(t+\tfrac{T}{2}-u\big)\,\mathrm{d}u = -\int_0^\infty h(u)\,v(t-u)\,\mathrm{d}u = -\,i(t). \tag{13}$$

The response of *any* linear circuit is therefore forced to be antisymmetric, irrespective of how many elements it contains; equivalently, its Fourier series contains only odd multiples of the

drive frequency. We verified Equation 13 numerically for all four topologies: the relative residual asymmetry $\max_t |i(t + T/2) + i(t)| / \max_t |i(t)|$ is 1.1×10^{-12}, i.e. at machine precision (Figure S4g).

This expectation can be tested directly on the data. Assigning each half-period to a rising or a falling edge from the sign of its fitted transient amplitude, the Group A and Group B solvents divide roughly evenly between the two, as a linear response requires: four and four for *n*-decane, anisole and 1,2,4-trichlorobenzene, five and three for 1,2-dichlorobenzene, two and six for mesitylene, whose transient is in any case at the noise floor. For Group C the division fails outright. In γ-butyrolactone and in propylene carbonate *all eight* retained half-periods carry a transient of the same sign, and in γ-valerolactone six of eight do. The half-wave antisymmetry required of any linear network is therefore violated by the measurements themselves, and not only by comparison with a simulated circuit.

The pronounced asymmetry observed in Group C is therefore not evidence that the adopted circuit is too simple: it excludes every linear circuit, and adding elements would yield fitted parameters without physical meaning. Replacing the ideal interfacial capacitance by a field-dependent one,

$$C(V) = \frac{C_{\mathsf{eq}}}{1 + (V/V_{\mathsf{sat}})^2}, \tag{14}$$

breaks the symmetry at once, the asymmetry growing from 2.6×10^{-7} at $V_{\mathsf{sat}} = 10$ V to 1.1×10^{-3} at 0.8 V and 4.9×10^{-2} at 0.4 V as saturation sets in within the experimental bias range (Figure S4g, right). This is why the Group C response is analysed in the main text through its physical mechanism — field-induced dipolar ordering — rather than through an expanded equivalent circuit.

S4.3 Re-extraction of the circuit parameters

Prompted by the reviewers' queries on the magnitude of the fitted capacitances and on the reliability of the extracted time constants, the whole analysis chain was rebuilt and re-run from the raw traces. The procedure is unchanged in substance — point-by-point median across traces, linear detrending, segmentation into half-periods, Theil–Sen estimation on the return map — and differs in three respects, each of which is a correction rather than a change of method.

Voltages are measured, not assumed. For every half-period the excitation waveform is read from the acquired voltage channel, and two quantities are recorded: the plateau, V_{plateau}, and the step at the leading edge, ΔV_{edge}. The interfacial transient is driven by the step, because in periodic steady state the interfacial capacitance is charged to $-V_0$ immediately before the edge and the voltage across R_s at $t = 0^+$ is therefore $V_0 - (-V_0) = 2V_0$, whereas the plateau current is set by the instantaneous bias. Accordingly

$$R_s = \frac{|\Delta V_{\text{edge}}|}{|A|}, \qquad R_m = \frac{|V_{\text{plateau}}|}{|B|}, \qquad C_{\text{eq}} = \frac{\tau}{R_s}. \tag{15}$$

Measuring the step rather than assuming it also handles the first driven half-period correctly: there the excitation starts from the DC set-point bias of approximately 0.2 V rather than from $-V_0$, and the measured step is approximately 1.2 V instead of 2 V.

Segment selection. Each 100 ms acquisition contains ten 10 ms segments. The first and the last are held at the DC set-point bias rather than driven by the square wave, and are excluded from the averages.

Rising and falling edges are identified from the data. Each half-period is assigned to a rising or a falling edge from the sign of the fitted transient amplitude, rather than from the parity of its index. This turns out to carry information in its own right, as discussed in Section S4.2.5.

The complete post-processing chain, from the raw traces to the fitted parameters and the figures built from them, is available at `https://github.com/Molecular-Electronics-Liverpool/NanoEDL`, organised as one directory per analysis step so that every intermediate product can be inspected.

S5 Absence of a mains contribution at 50 Hz

The excitation frequency used throughout this work coincides with the European mains frequency, and we therefore tested for a mains contribution directly rather than relying on shielding and detrending alone.

S5.1 Orthogonality of the mains to the excitation

A square wave of frequency f_d and amplitude V_0 has the Fourier series

$$v(t) \;=\; \frac{4V_0}{\pi} \sum_{n \text{ odd}} \frac{1}{n}\, \sin(2\pi n f_\mathrm{d} t)\,, \tag{16}$$

that is, odd harmonics only. Over a record of length T_R two sinusoids at f and f' are exactly orthogonal whenever fT_R and $f'T_\mathrm{R}$ are distinct integers,

$$\frac{2}{T_\mathrm{R}} \int_0^{T_\mathrm{R}} \sin(2\pi f t)\, \sin(2\pi f' t)\, \mathrm{d}t \;=\; \delta_{ff'}. \tag{17}$$

Our records are 100 ms long, so the orthogonal grid has 10 Hz spacing. In the 100 Hz control dataset the excitation occupies 100, 300, 500 Hz and so on, whereas the mains frequency satisfies $f_\mathrm{mains}T_\mathrm{R} = 5 \in \mathbb{Z}$ and is not an odd multiple of 100 Hz. By Equation 17 the 50 Hz projection is exactly free of leakage from the drive, so whatever amplitude appears there is mains pickup and nothing else.

Projecting the median current of the 100 Hz anisole dataset (600 traces) onto that grid gives a drive line of 448 pA at 100 Hz and a 50 Hz amplitude of 1.6 pA (Figure S5). The median amplitude across all non-drive bins up to 1 kHz is 3.5 pA and their 95th percentile is 53 pA: the 50 Hz line falls at the 26th percentile of that distribution, i.e. it is among the quietest bins in the whole spectrum. In absolute terms it is 0.4 % of the drive and more than an order of magnitude below the instrumental noise floor, against an interfacial transient in anisole spanning approximately 1.4 µA.

S5.2 Rejection of an incoherent component by the median

The square-wave excitation is generated by the PXI source and is not phase-locked to the mains, so the pickup contributed by trace k is $p_k(t) = P \sin(2\pi f_\mathrm{mains} t + \varphi_k)$ with phases φ_k independent and uniform on $[0, 2\pi)$. At any fixed t the $p_k(t)$ are independent draws from a symmetric arcsine distribution of zero median, and the point-by-point median across N traces converges to zero with standard error

$$\mathrm{SE}\big[\, \mathrm{med}_k\, p_k(t) \,\big] \;=\; \frac{1}{2 f_p(0)\sqrt{N}} \;=\; \frac{\pi P}{2\sqrt{N}}, \tag{18}$$

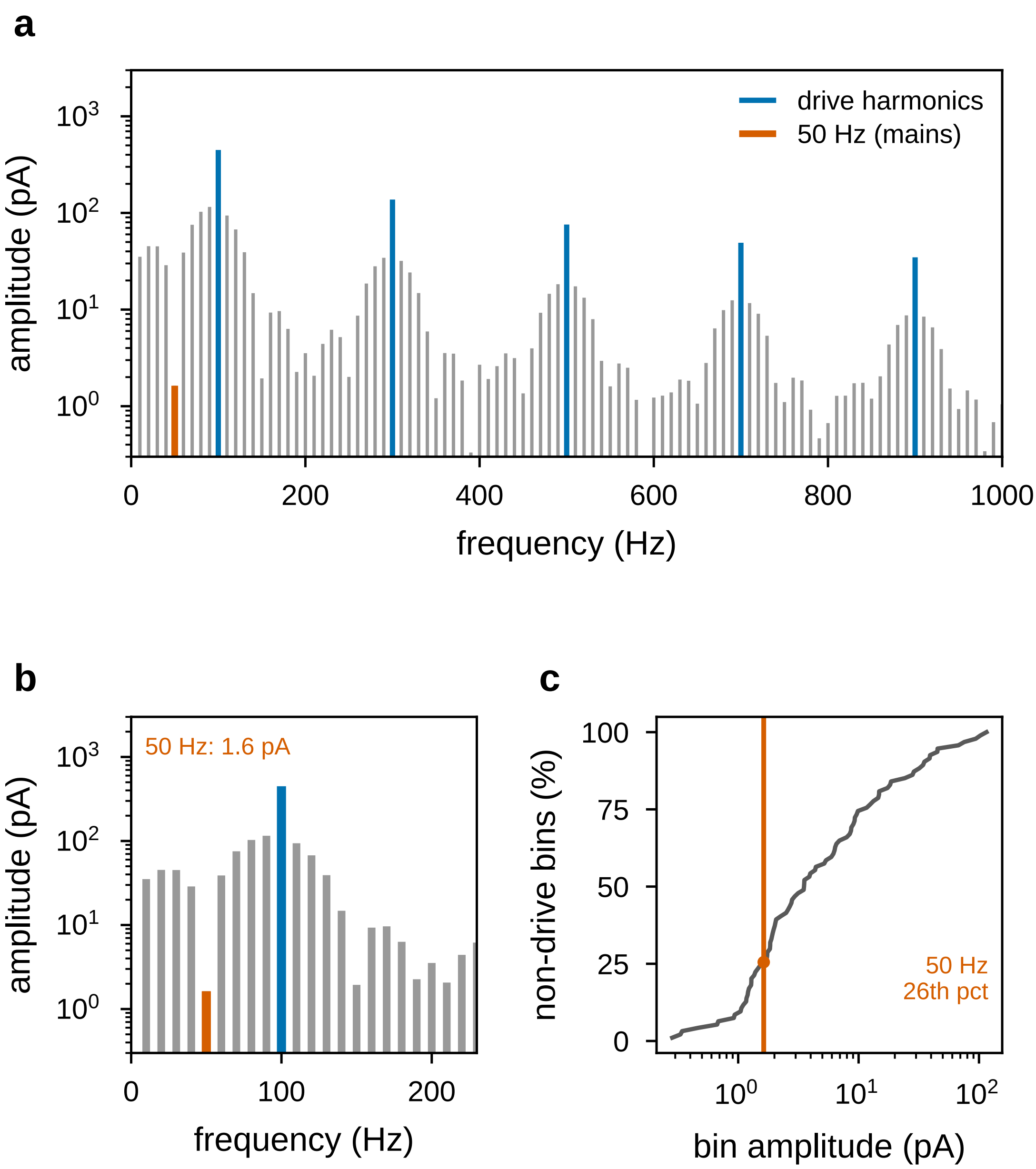

Figure S5: Leakage-free search for a mains component. (a) Amplitude spectrum of the median current of the 100 Hz anisole dataset on the orthogonal 10 Hz grid. The odd harmonics of the excitation (Equation 16) are marked in blue; 50 Hz, which is orthogonal to the drive and therefore reports the mains alone, is marked in red. (b) Detail below 220 Hz. (c) Cumulative distribution of the amplitudes of all non-drive bins, with the 50 Hz value marked: it falls at the 26th percentile, i.e. it is statistically unremarkable among bins where no signal is expected.

where $f_p(0) = 1/(\pi P)$ is the density of the arcsine law at the origin. With the trace counts of Table S4 (N between 884 and 8936) Equation 18 gives one to two orders of magnitude of rejection on top of the shielding. This is a property of the acquisition scheme rather than an assumption about it.

S5.3 Further controls

Three observations reported in the main text bear on the same question. Group A solvents were measured under an identical 50 Hz excitation and show no transient at all, whereas a mains artefact would be independent of the solvent in the cell. Reducing the drive amplitude from $2\,V_{pp}$ to $0.2\,V_{pp}$ suppresses the transient, whereas mains pickup does not scale with our drive amplitude. Finally, the response changes qualitatively between 50 and 100 Hz, as expected for a relaxation process with a finite time constant. Taken together with the spectral measurement above, the data already in hand settle the question.

S6 Validation of the extraction against a network of known composition

The time constants reported here are of the order of 0.1 ms to 0.7 ms, the shortest of which corresponds to only three to four samples at our 40 kHz sampling rate. We therefore calibrated the whole acquisition and extraction chain against a network whose components are known independently.

The STM cell was replaced, at the head of the instrument, by a dummy cell comprising a $910\,k\Omega$ resistor in series with a 100 pF capacitor, with the resistor box left at its usual $1\,k\Omega$ (Figure S6a). The nominal time constant is $\tau = (R + R_{Box})C = 91.1$ μs, that is 3.6 samples per time constant — deliberately shorter than every time constant reported in this work for a solvent that shows a resolvable transient. The cell was driven with the same square-wave excitation and acquired with the same instrument and settings as a molecular junction. The record was divided at the switching edges, the point-by-point median was taken over all $42\,075$ transients, and a single exponential relaxing onto a constant baseline was fitted to it, exactly as for junction data.

The fit returns $\tau = 91.45 \pm 0.23$ μs against the 91.1 μs expected from the nominal components, an agreement of 0.4 %, corresponding to a recovered capacitance of 100.4 pF against 100 pF.

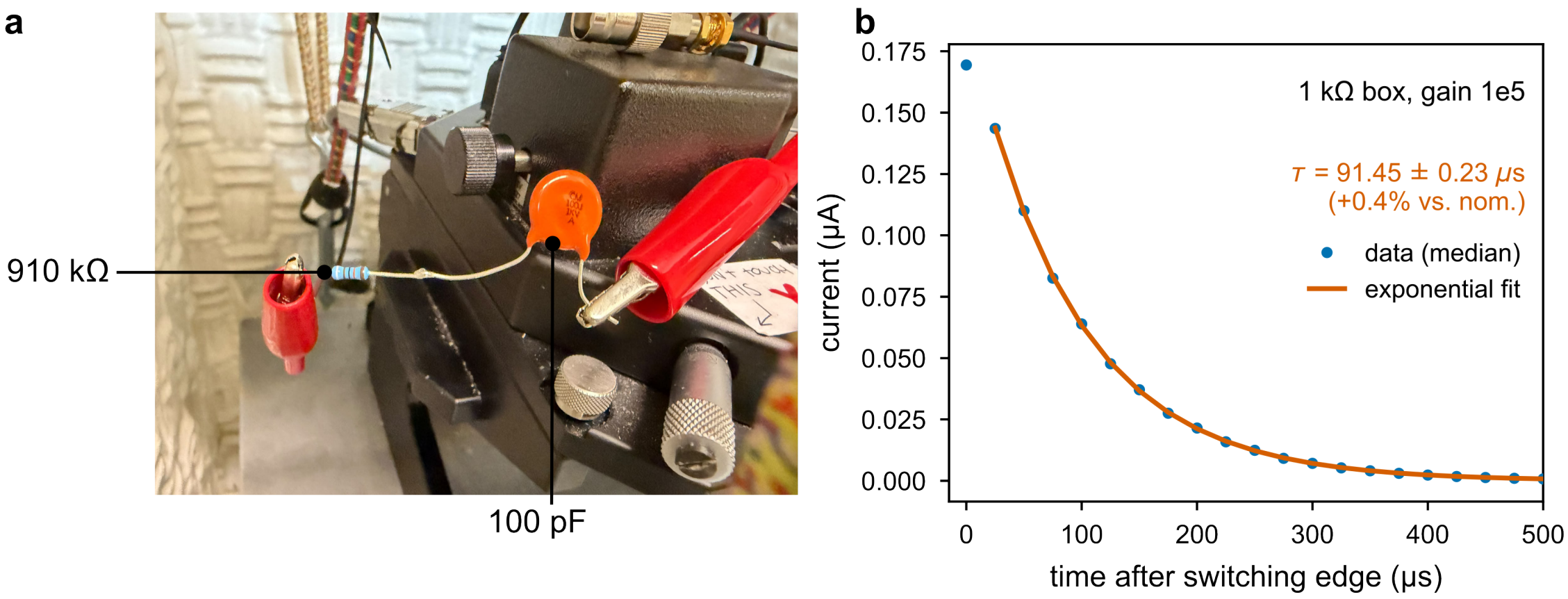

Figure S6: Validation of the extraction against a network of known composition, demonstrating that the measured branch is shielded from the parasitic capacitance of the cabling and the instrument. (a) The dummy cell in place at the head of the instrument: a $910\,\mathrm{k\Omega}$ resistor in series with a 100 pF capacitor, connected in place of the STM cell. (b) Point-by-point median of the measured switching transients (blue) and the single-exponential fit (orange); the shaded band marks the samples excluded because the excitation has not yet settled. The fit returns $\tau = 91.45 \pm 0.23$ μs against the 91.1 μs expected from the nominal components, that is a recovered capacitance of 100.4 pF against a 100 pF standard. The cabling and the resistor box contribute some 700 pF of parasitic capacitance in total (Section S3.1); had any of it lain in the measured branch, the recovered value would have exceeded the nominal one by roughly a factor of eight, so this measurement bounds the parasitic contribution to the fitted C_{eq} well below 1 %.

Two consequences follow. First, at 3.6 samples per time constant the extraction is accurate to better than 1 %, so the time constants reported here for Group B and Group C, all of which are at least three times longer, are not artefacts of the sampling rate. The Group A entries of Table S5 are shorter, but those solvents show no resolvable transient and those values are fits to noise. Second, the parasitic capacitance of the cabling and the resistor box quantified in Section S3.1 does not enter the measured branch: were the 700 pF of C_{tot} in series with the cell, a 100 pF standard could not have been recovered to 0.4 %. That capacitance sits at a node held at virtual ground by the transimpedance amplifier and is therefore invisible to C_{eq}.

The same measurement identifies the one systematic that does matter, which is the settling of the excitation rather than the sampling rate. In a first acquisition with the resistor box set to $47\,\mathrm{k\Omega}$, charging the cable capacitance through the larger series resistance made the drive take approximately 300 μs — some three time constants — to reach its plateau, and a fit started at the switching edge returned τ up to 35 % high, because it was measuring the excitation rather than the relaxation. Returning the resistor box to the $1\,\mathrm{k\Omega}$ used throughout this work shortens the settling to a single sample and removes the bias. All junction data reported here were acquired in that configuration, and the fit windows exclude the settling interval.

Finally, because the dummy cell is linear by construction, any departure it shows from $i(t + T/2) = -i(t)$ is instrumental rather than physical and therefore sets the experimental floor for the half-wave asymmetry discussed in Section S4.2.5. Measured over $15\,976$ complete drive periods, that floor is 5×10^{-4}, far below the asymmetry observed in Group C.

References

(1) Naher, M.; Bock, S.; Langtry, Z. M.; O'Malley, K. M.; Sobolev, A. N.; Skelton, B. W.; Korb, M.; Low, P. J. Synthesis, Structure and Physical Properties of “Wire-like” Metal Complexes. *Organometallics* **2020**, *39*, 4667–4687.

(2) *CRC Handbook of Chemistry and Physics*, 97th ed.; Haynes, W. M., Ed.; CRC Press/-Taylor & Francis: Boca Raton, FL, 2016.

(3) Al-Kandary, J. A.; Al-Jimaz, A. S.; Abdul-Latif, A.-H. M. Viscosities, Densities, and Speeds of Sound of Binary Mixtures of Benzene, Toluene, *o*-Xylene, *m*-Xylene, *p*-Xylene, and Mesitylene with Anisole at (288.15, 293.15, 298.15, and 303.15) K. *Journal of Chemical & Engineering Data* **2006**, *51*, 2074–2082.

(4) Desyatnyk, O.; Pszczółkowski, L.; Thorwirth, S.; Krygowski, T. M.; Kisiel, Z. The Rotational Spectra, Electric Dipole Moments and Molecular Structures of Anisole and Benzaldehyde. *Physical Chemistry Chemical Physics* **2005**, *7*, 1708–1715.

(5) Sabzekar, M.; Pourafshari Chenar, M.; Khayet, M.; García-Payo, C.; Mortazavi, S. M.; Golmohammadi, M. Development of Novel Electrospun Fibers Based on Cyclic Olefin Polymer. *Nanomaterials* **2023**, *13*, 2412.

(6) Bhatia, S. C.; Sangwan, J.; Rani, R.; Bhatia, R. Densities, Viscosities, Speeds of Sound, and Refractive Indices of Binary Mixtures of 2-Octanol with Chlorobenzenes. *International Journal of Thermophysics* **2011**, *32*, 2027–2039.

(7) Fornefeld-Schwarz, U. M.; Svejda, P. Refractive Indices and Relative Permittivities of Liquid Mixtures of γ-Butyrolactone, γ-Valerolactone, δ-Valerolactone, or ε-Caprolactone + Benzene, + Toluene, or + Ethylbenzene at 293.15 K and 313.15 K and Atmospheric Pressure. *Journal of Chemical & Engineering Data* **1999**, *44*, 597–604.

(8) Papović, S.; Gadžurić, S.; Tot, A.; Bešter-Rogač, M.; Pavlović, K.; Vraneš, M. A Comparative Study on the Interactions of [bmim][NTf_2] Ionic Liquid with Selected Four- to Seven-Membered-Ring Lactones. *The Journal of Chemical Thermodynamics* **2017**, *107*, 170–181.

(9) Krakowiak, J.; Śmiechowski, M. Excess Molar Volume and Viscosity Deviation for Binary Mixtures of γ-Butyrolactone with Dimethyl Sulfoxide. *The Journal of Chemical Thermodynamics* **2017**, *110*, 57–64.

(10) Wankhede, D. S.; Wankhede, N. N.; Arbad, B. R.; Lande, M. K. Viscosity Behavior of Some Oxygen Containing Compounds. *International Journal of Thermophysics* **2010**, *31*, 2239–2245.

(11) Wu, C.; Qiao, X.; Robertson, C. M.; Higgins, S. J.; Cai, C.; Nichols, R. J.; Vezzoli, A. A Chemically Soldered Polyoxometalate Single-Molecule Transistor. *Angewandte Chemie International Edition* **2020**, *59*, 12029–12034.

(12) Naghibi, S.; Sangtarash, S.; Kumar, V. J.; Wu, J.-Z.; Judd, M. M.; Qiao, X.; Gorenskaia, E.; Higgins, S. J.; Cox, N.; Nichols, R. J.; Sadeghi, H.; Low, P. J.; Vezzoli, A. Redox-Addressable Single-Molecule Junctions Incorporating a Persistent Organic Radical**. *Angewandte Chemie International Edition* **2022**, *61*, e202116985.

(13) Daaoub, A.; Morris, J. M. F.; Béland, V. A.; Demay-Drouhard, P.; Hussein, A.; Higgins, S. J.; Sadeghi, H.; Nichols, R. J.; Vezzoli, A.; Baumgartner, T.; Sangtarash, S. Not So Innocent After All: Interfacial Chemistry Determines Charge-Transport Efficiency in Single-Molecule Junctions. *Angewandte Chemie International Edition* **2023**, *62*, e202302150.

(14) Morris, J. M. F.; Potter, J.; Gorenskaia, E.; Abram, R. T.; Naher, M.; Spano, C. E.; Dixon, E. L.; Sil, A.; Rousset, E.; Higgins, S. J.; Nichols, R. J.; Low, P. J.; Vezzoli, A. Charge Transport through Linear Carbon Atomic Chains, en, 2025.

(15) Sil, A.; Spano, C. E.; Chelli, Y.; Higgins, S. J.; Sangtarash, S.; Piccinini, G.; Graziano, M.; Nichols, R. J.; Sadeghi, H.; Vezzoli, A. Single-Molecule Mechanoresistivity by Intermetallic Bonding. *Angewandte Chemie International Edition* **2025**, *64*, e202418062.

(16) Sen, P. K. Estimates of the Regression Coefficient Based on Kendall's Tau. *Journal of the American Statistical Association* **1968**, *63*, 11.

(17) Morris, J. M. F.; Potter, J.; Bates, D.; Wu, C.; Robertson, C. M.; Higgins, S. J.; Nichols, R. J.; Low, P. J.; Vezzoli, A. Digging through the (Statistical) Dirt: A Reproducible Method for Single-Molecule Flicker Noise Analysis. *The Journal of Physical Chemistry C* **2025**, *129*, 4097–4104.

(18) Galleani, L. Response of Dynamical Systems to Nonstationary Inputs. *IEEE Transactions on Signal Processing* **2012**, *60*, 5775–5786.
